# Multi-meta-omics reveal unique symbiotic synchronization between ectomycorrhizal fungus and soil microbiome in *Tricholoma matsutake* habitat

**DOI:** 10.1186/s40168-025-02292-7

**Published:** 2025-12-11

**Authors:** In Hyup Bae, Hyun Kim, Su-Min Kim, Yong-Hwan Lee

**Affiliations:** 1 Department of Agricultural Biotechnology, Seoul National University, Seoul, 08826 Republic of Korea; 2 Research Institute of Agriculture and Life Sciences, Seoul National University, Seoul, 08826 Republic of Korea; 3 Interdisciplinary Programs in Agricultural Genomics, Seoul National University, Seoul, 08826 Republic of Korea; 4 Plant Immunity Research Center, Center for Plant Microbiome Research, and Plant Genomics and Breeding Institute, Seoul National University, Seoul, 08826 Republic of Korea

**Keywords:** Metatranscriptomics, Metagenomics, *Tricholoma matsutake*, Mycorrhizal symbiosis, Metabolic synchronization

## Abstract

**Background:**

Ectomycorrhizal (ECM) fungi establish symbiotic relationships with plant roots, enhancing nutrient uptake, improving plant health, and boosting ecosystem resilience. Although previous studies reported molecular interactions among plant-ECM fungi-surrounding microbes near plant roots, microbiome-wide metabolic shifts and associations with the fungi remain unclear.

**Results:**

Using *Tricholoma matsutake* as a model, we initially found that *T*. *matsutake* induced remarkable microbial community turnover linked to altered soil moisture, nitrogen, and phosphorus levels. Parallel with the compositional alteration, microbiome-wide metabolic capacities, including glutamate metabolism, oligopeptide transport, and siderophore activity, were enriched in the *T*. *matsutake*-colonizing soil compared to the soils where the fungus was not colonized. From metatranscriptome data, we found that *T*.* matsutake* induced functional remodeling in nitrogen metabolism. Notably, the fungus and soil microbiome were metabolically synchronized with the upregulation of nitrate reduction, glutamate biosynthesis, tryptophan biosynthesis, and indole-3-acetic acid (IAA) biosynthesis. Metabarcoding and metatranscriptome-guided microbial associations revealed potential *T*. *matsutake* helper bacteria consisting of *Conexibacter* and *Paraburkholderia*. Phage community analyses further showed that the colonization of the ECM fungus influenced phage distributions along with the increase in temperate phage populations. The differential expression of auxiliary metabolic genes also demonstrated that phages could influence bacterial fitness in response to *T*. *matsutake* colonization.

**Conclusion:**

Our multi-meta-omics-based approaches revealed unique environmental changes by *T*. *matsutake* compared to other mycorrhizal systems, as well as metabolic synchronization between the ECM fungus and surrounding microbiomes. These findings will expand our understanding of ECM symbiotic frameworks by highlighting integrated microbial and viral metabolic dynamics.

Video Abstract

**Supplementary Information:**

The online version contains supplementary material available at 10.1186/s40168-025-02292-7.

## Background

Choice edible fungi are considered essential foods due to their high nutritional and culinary values. Approximately 2000 fungal species, including truffles (*Tuber* spp.) and pine mushrooms (*Tricholoma matsutake*; hereafter *Tm*), were consumed as food and medicinal sources worldwide [1]. Among them, only 35 fungi are commercially cultivated under controlled conditions [2], indicating that the production of remaining fungi highly relies on environmental conditions in nature. To increase the production stability of non-cultivable fungi, researchers have endeavored to understand the biology of the fungi and construct artificial cultivation systems based on these understandings. One of the edible fungi that has been widely studied in this context is truffles. The development of reliable inoculation techniques in the 1960s enabled widespread plantation establishment [3]. With this practical effort, to understand the ecology of truffles, the genomic features of them were extensively unraveled in the 2010s [4]. The structure and roles of microbes in or on truffles have also been thoroughly studied [5–7], revealing that they influence aroma formation in truffles [8]. In contrast, *T*. *matsutake* is a culturally and economically important ectomycorrhiza (ECM) species in East Asia, yet it remains entirely dependent on wild harvests [3, 9]. Despite its comparable ecological role and gastronomic value, efforts toward artificial cultivation have achieved only limited success [3]. This knowledge gap between truffles and *T*. *matsutake* highlights the critical need for focused research.

*Tm* is commonly found in East Asia and Europe, forming obligate ECM associations with *Pinus densiflora*, especially in Japan and the Korean peninsula [9–11]. It also develops a specialized underground structure known as the shiro, a dense mycelial network in the soil that interfaces with host roots [12]. Within this structure, the Hartig net is formed at the mycorrhizal interface to facilitate nutrient exchange [13]. Over the past century, the abundance of matsutake mushrooms has declined drastically due to forest succession, pine wilt disease, and changing land management practices [14]. Despite a longstanding interest in artificial cultivation, human-mediated reproductive growth of *Tm* in controlled conditions is still elusive [15]. *Tm* remains one of the most commercially valuable yet least cultivable choice edible fungi.

Mycorrhizal symbioses have traditionally been viewed as binary interactions between the mycorrhizal fungus and its host plant. However, this paradigm is shifting as additional third-party partners are increasingly recognized. Among mycorrhizal interactions, bacterial associates, commonly referred to as mycorrhizal helper bacteria (MHB), have been shown to promote colonization and nutrient cycling [16, 17]. A well-characterized example within ECM symbiosis is *Pseudomonas fluorescens* BBc6R8, which promotes the establishment of symbiosis between *Laccaria bicolor* and Douglas fir by priming fungal growth, altering hyphal morphology, and modulating fungal gene expression during early interaction stages [18]. A recent finding further suggested that fungal-derived auxins, particularly indole-3-acetic acid (IAA), may contribute to ECM symbiosis [19].

Recent reports show that mycorrhizal hyphae serve as unique ecological habitats providing inhabiting spaces and releasing selective exudates [20, 21]. It has also been suggested that certain fungus-associated bacteria exhibit symbiotic adaptations associated with ECM hyphae [22], highlighting the active role of fungal hyphae in shaping microbial partnerships. These findings shift the traditional concepts of ECM systems, ecological contributors connecting host plants and soil, to dynamic multipartite networks actively regulating underground multi-kingdom interactions [23, 24]. Thus, understanding how ECM shapes these unique interactions is a critical cornerstone, with implications for mycorrhizal biology and ecosystem functioning.

Previous studies on microbial communities in the *Tm* habitat, which refers to the forest soil environments where *Tm* establishes ectomycorrhizal associations with host trees, predominantly *P*. *densiflora*, reported the compositional differences in soil microbial communities between *Tm*-dominant condition (or shiro-forming soil) and *Tm*-minor condition (non-shiro-forming soil) [25, 26]. Using a metabarcoding-based survey and microbial network analyses, putative *Tm*-associated bacterial (i.e., *Bradyrhizobium*, *Mycobacterium*, and *Burkholderia*-*Caballeronia*-*Paraburkholderia*) and fungal taxa (i.e., *Umbelopsis*, *Oidiodendron*, *Fusarium*, and *Trichoderma*) were proposed [27, 28]. Until now, however, functional or metabolic associations between *Tm* and soil bacterial and fungal communities have not been profoundly understood.

Here, we aimed to answer the following questions: (i) What abiotic factors are changed by *Tm* dominance? (ii) What abiotic factors are linked with microbial community-level compositional turnover? (iii) How are community-wide microbial functions altered with *Tm* colonization? (iv) What microbes are functionally or metabolically associated with *Tm*? And (v) What roles do auxiliary microorganisms, such as phages, play in the *Tm* ecosystem? To address these questions, we conducted multi-omic analyses of soil microbiomes within the *Tm* habitat. By integrating metagenomic and metatranscriptomic data, we captured both the taxonomic composition and gene expression profiles of the microbial communities. Our approach applied high-resolution comparative analysis to uncover community-wide functional patterns associated with *Tm* reproduction. By resolving comprehensive (DNA-level) and active (RNA-level) microbial activity, we identified ecological factors that may support or modulate the complex ECM association. These findings offer a new framework for understanding *Tm* ecology and highlight the potential role of multipartite interactions in sustaining ECM symbioses.

## Methods

### Soil sampling

To investigate the spatial and temporal dynamics of soil microbial communities in a natural *Tm* habitat, soil samples were collected from the experimental forest of the National Institute of Forest Science in Hongcheon-gun (37° 41′ 14.8′′N 128° 00′ 20.5′′E), Republic of Korea (Fig. S1a, b). The vegetation in the sampling area consisted of *P*. *densiflora*, *Rhododendron mucronulatum*, *Quecus mongolica*, *Styrax obassia*, *Elaeagnus umbellata*, and *Lindera obtusiloba* (Fig. S1c). We selected a large, stable fairy ring (5.55 m horizontally × 5.26 m vertically) where fruiting bodies had been consistently observed for several years (Fig. S1c). This ensured that the site represented a mature and persistent mycorrhizal system, suitable for repeated sampling. Since *Tm* mycelia typically expand outward at a rate of ~ 10 cm per year [29], we designed a sampling scheme that captured this ecological scale of growth. The colonization of *Tm* was confirmed by observing the presence of *Tm* mycelia on the roots of *P*. *densiflora* and fairy rings in soils (Fig. S1d, e). Direct sampling beneath the visible fairy ring was avoided, as previous studies have shown this soil to be overwhelmingly dominated by *Tm* sequences [26, 30], obscuring the broader microbial community. Instead, we established two transects on the west side of the ring (Fig. S1c). Along the inner transect (inside the ring), we collected five “*Tm*-dominant” (TmD) samples beginning 10 cm inward from the ring edge, with subsequent samples at 10 cm intervals (+ 1 to + 5; up to 50 cm). Along the outer transect (outside the ring), we collected five “*Tm*-minor” (TmM) samples beginning 20 cm outward from the ring edge, with subsequent samples at 10 cm intervals (− 1 to − 5; up to 60 cm). This design enabled us to contrast fungal-dominated versus less-colonized soils while keeping spatial structure consistent. To capture seasonal variation, soil was collected every 2 months at five time points in March, May, July, September, and November of 2022. When collecting samples, the leaf litter layer was removed first, and clean soil was collected by digging a 7 cm × 7 cm hole at a depth of 10 cm using a trowel. Approximately 300 g of soil was obtained. The collected soil was then sealed in a zip-lock bag. Every sampling spot was flagged with an identification tag. A total of 25 TmD soil samples and 25 TmM soil samples were collected. About 50 g of each sample was stored in a − 70 °C deep freezer for amplicon-based sequencing, and the remaining soil was stored in a 4 °C cold room. Soil DNA extraction was completed within two weeks of soil sampling. About 220 g of TmD (+ 1 to + 3) and TmM (− 3 to − 5) soils collected in July, September, and November were used for soil chemical analysis. In September 2023, soil samples were collected using the same procedure as in 2022, with a modification for RNA extraction. For each 2023 sampling spot, duplicate soil samples were collected simultaneously: one for DNA extraction (metagenomic analysis) and one for RNA extraction (metatranscriptomic analysis). Soil samples for RNA extraction were transferred into sterile 50 mL conical tubes. The tubes were immediately submerged in liquid nitrogen within a 4 L portable Dewar flask (model KG.1214; KGW Isotherm, Karlsruhe, Germany) to stabilize RNA during field collection and transport. Soil samples for DNA extraction were treated and transported in the same manner as in 2022. Upon arrival at the laboratory, all soil samples were stored in a − 70 °C deep freezer until extraction.

### Soil chemistry

To investigate the effects of *T*. *matsutake* colonization on soil chemical environments, we examined soil texture (contents of sand, silt, and clay) and chemical properties (pH, soil organic matters [SOM], total nitrogen [TN], total phosphorous [TP], exchangeable Ca^2+^, Mg^2+^, Na^+^, K^+^, Fe, Mn, Cu, Zn, phosphate [P_2_O_5_], and soil water content). A total of 300 g of each soil, which was collected from July (*n* = 6; three samples for TmD and TmM, respectively), September (*n* = 6), and November (*n* = 6) in 2022, was used to analyze them. Detailed methods for investigating the soil chemical properties are described in Method S1 of the Supporting Information.

### DNA extraction and library preparation of metabarcoding data

To investigate the bacterial and fungal communities from TmD and TmM soils using metabarcoding data, 0.5 g of each soil sample was transferred to a Lysing Matrix E tube and ground using a homogenizer (MP Biomedicals, Santa Ana, United States). DNA was extracted using the MagBeads FastDNA™ Kit for Soil (MP Biomedicals) following the manufacturer’s instructions. DNA concentrations were quantified using a NanoDrop™ spectrophotometer (Thermo Fisher Scientific, Waltham, United States). Extracted DNA was stored at − 20 °C. 16S and internal transcribed spacer (ITS) ribosomal RNA (rRNA) gene amplicons were generated by a two-step PCR amplification protocol (Method S2). Sequencing was conducted in the National Instrumentation Center for Environmental Management at Seoul National University.

### Metabarcode sequencing and processing

Sequenced reads were processed using QIIME2 (v. 2022.11) [31]. After demultiplexing, the 16S sequences were merged and quality-filtered with the options *–p-trunc-len-f 292 –p-trim-left-f 19 –p-trunc-len-r 257 –p-trim-left-r 20* using the DADA2plugin [32] in the QIIME2 pipeline. The lengths for truncation (*–p-trunc-len-f* and *–p-trunc-len-r*) were determined by checking the base position where the median quality score drops below 30. Meanwhile, the ITS reads were quality-filtered using the options *–p-trunc-len-f 0, –p-trim-left-f 22, –p-trunc-len-r 0, and –p-trim-left-r 20*. Truncation was not applied for the ITS reads because the large length variation in them may result in the loss of valid sequences. The processed reads were classified as amplicon sequence variants (ASVs). The taxonomies of the non-chimeric ASVs were assigned using the Naïve Bayes algorithm implemented in the q2-feature-classifier [33] based on the Silva database (v. 138) [34] for the V4 region of the 16S rRNA gene. For the ITS regions, taxonomic assignment was conducted using the q2-feature-classifier based on the UNITE database (UNITE_ver9_dynamic of October 2022 [35]. Bacterial sequences of > 300 bp and fungal sequences of < 100 bp were eliminated. Samples with low sequencing depth (< 1000 reads) were removed from the datasets. The ASV table was imported into R using the phyloseq package. Unwanted taxa were removed (for bacterial ASV: orders Chloroplast and Rickettsiales; for fungal ASV: kingdoms Unassigned, Chromista, and Plantae).

### Statistical analyses of metabarcoding data

Unless otherwise stated, statistical analyses were performed using R (v. 4.3.3) [36]. The threshold for statistical significance was ɑ = 0.05; where appropriate, statistical significance was corrected for multiple hypothesis testing by the false discovery rate method. For metabarcoding data, the ASV table was normalized by cumulative-sum scaling and log-transformed by the function *cumNorm* in the R package metagenomeSeq (v. 3.8) [37] to calculate beta diversity. Taxa with a relative abundance of > 0.5% were visualized using the R package ggplot2 (v. 3.2.1) [38] for taxonomic composition analysis. Alpha diversity of the bacterial and fungal communities was determined using the function *alpha* implemented in the R package microbiome (v. 1.24.0) [39]. Ordination analyses and two-way permutational analysis of variance (two-way PERMANOVA) were conducted using the function *ordinate* in the R package phyloseq (v. 1.46.0) [40] and the function *adonis2* in the R package vegan (v. 2.6–8) [41], respectively. To find correlations between soil chemical properties and community diversity, the function *mantel* implemented in the R package vegan was used.

### Acquisition of metagenomic data

The genomic DNA was extracted from the soil samples collected in September 2022 (*n* = 10) and 2023 (*n* = 10) using SPINeasy® DNA Pro Kit for Soil (MP Biomedicals, CA, USA) following the manufacturer’s instructions with a few modifications (Method S3). Triple technical replicates of genomic DNA were extracted from each soil sample, so a total of 30 samples (10 soil samples per year × three technical repeats per soil) were prepared, and the replicate with the highest DNA Integrity Number (DIN) and total amount was selected for the library construction. The libraries of a total of 20 whole metagenome samples were constructed and sequenced from Macrogen (Seoul, Republic of Korea) using TruSeq Nano DNA Library Prep Kit (Illumina, CA, USA) and the NovaSeq 6000 System (Illumina, CA, USA), respectively (Method S3).

The quality of raw metagenomic reads was checked using FastQC (v. 0.12.1) [42] prior to trimming. The sequenced raw reads were trimmed using Trimmomatic (v. 0.39) [43] with the options *leading 3*, *trailing 3*, *sliding window 4:15*, and *minlen 36*. The quality of the trimmed reads was checked once again with FastQC. To recover microbial contigs from the metagenomic data, trimmed and quality-checked reads were assembled in two ways: individual assembly and co-assembly. We respectively co-assembled metagenomic reads obtained from *Tm*-dominant and minor sites to minimize the possibility of getting chimeric contigs. For both strategies, we used megahit (v. 1.2.9) [44] with the options *–presets meta-large* and *–min-contig-len 300*. Other default options were used for the assembly. The quality of metagenomic assemblies was assessed using stat.sh implemented in BBTools (v. 39.03) [45]. The resulting contigs were taxonomically classified using Diamond (v. 2.1.8) [46] with the NCBI nr DB (assessed on October 25, 2023). Based on the classification results, bacterial and fungal contigs were obtained.

### Metagenomic gene annotation and clustering

Putative bacterial and fungal genes were structurally annotated from the obtained bacterial and fungal contigs using MetaProkka (modified version of Prokka [47]; v. 1.15.0; https://github.com/telatin/metaprokka) and MetaEuk (v. 6.a5d39d9) [48], respectively. Default options were used for the annotation. After the structural annotation, a total of 40,440,240 bacterial and 1,224,896 fungal genes (proteins) were predicted. The predicted proteins were further clustered into representatives using mmseqs2 (v. 15.6f452) [49] with the default option, resulting in 17,024,354 bacterial and 579,346 fungal representative proteins. The orthologs of the representative proteins were searched using emapper (v. 2.1.12) [50] with EggNOG DB (v. 5.0.2) [51].

### Acquisition of metatranscriptomic data

For RNA extraction from the soil samples collected in September 2023 (*n* = 6; TmM5, TmM4, TmM1, TmD1, TmD4, and TmD5), the SPINeasy® DNA/RNA Kit for Soil (MP Biomedicals, Irvine, CA, USA) was utilized following the manufacturer’s instructions, with three modifications to optimize the protocol for low-biomass soils (Method S4). The libraries of a total of six metatranscriptomic samples were constructed and sequenced from Macrogen using the Illumina Stranded Total RNA Prep with Ribo-Zero Plus Microbiome kit (Illumina, San Diego, CA, USA) and the NovaSeq 6000 System, respectively (Method S4).

The initial quality of metatranscriptomic reads was checked with FastQC, and the reads were trimmed with Trimmomatic as described in the trimming of metagenomic reads. Any overrepresented sequences and G polymers identified by FastQC were removed using BBDuk (bbduk.sh) implemented in BBTools. From the qualified metatranscriptomic reads, ribosomal RNA reads were removed using SortMeRNA (v. 4.3.6) [52] with smr_v4.3 dbs and the options *–paired_out*. The resulting reads were used to quantify metagenomic gene expression levels.

### Quantification of gene abundance and expression levels

The abundance of the predicted genes was quantified by mapping metagenomic reads onto the DNA sequences of each gene using bwa (v. 0.7.17-r1188) [53]. Further quantification was performed using Salmon (v. 1.10.3) [54] with the option *-l ISU*. The mapped reads were normalized to RPKM and TPM, respectively. Meanwhile, the expression levels of the predicted genes were quantified by mapping metatranscriptomic reads onto the DNA sequences of each gene using bwa. Further quantification was performed using Salmon with the option *-l ISR* since the metatranscriptomic reads were obtained using a reverse-strand-specific manner. The mapped reads were normalized to RPKM and TPM, respectively.

### Statistical analyses of metagenomic and *metatranscriptomic* data

Metagenomic and metatranscriptomic data were also analyzed using R. Non-metric multidimensional scaling (NMDS) was performed using the R package phyloseq. The enrichment tests of functional features were conducted using DESeq2 (v. 1.42.1) [55]. For further analyses, we divided soil samples into three groups based on the *Tm* colonization status, determined by the incidence of *Tm* mycelia observed during sample collection, the fruiting body formation history of the sampling site, and the population distribution patterns available in the metagenomic data. The “Pre-colonization” (Pre) group refers to soils where *Tm* had not yet colonized; therefore, *Tm* mycelia were not observed in the soils, with no detectable population in the metagenomic data. The “Active-colonization” (Active) group is defined as soils where *Tm* mycelial colonization was visually observed (Fig. S1), and its population was also detected in the metagenomic data. Lastly, the “Post-colonization” (Post) group refers to soils where *Tm* mycelia had previously been observed, but its population was no longer detected in the metagenomic data. The analyses were conducted for three comparison pairs (Pre [*n* = 9] vs. Active [*n* = 4], Active vs. Post [*n* = 7], and Pre vs. Post). Differentially expressed genes were defined using the R package NOISeq (v. 2.46.0) [56], which could be applied when limited samples are available. Enriched Gene Ontology (GO) terms were determined using the R package topGO (v. 2.54.0) [57] with *Tm* status-specific DEGs (specifically upregulated genes in *Tm*-active sites; the detailed definition of *Tm* status-specific DEGs is available in Method S5). Metatranscriptomic gene expressions at the metabolic pathway level were assessed using the R package pathview (v. 1.42.0) [58]. Gene co-expression patterns and networks were assessed using the R package Mfuzz (v. 2.62.0) [59] and WGCNA (v. 1.73) [60], respectively, with correlation significance further examined by the SparCC algorithm implemented in FastSpar (v. 1.0.0) [61]. Detailed procedures, parameters, and statistical settings are described in Method S5.

### Comparative transcriptome analysis

To compare transcriptional differences between in vitro culture and soil conditions, the previously reported genome contigs of *Tm* (strain NIFoS 2001) were acquired from the NCBI Genome database (genome accession number: JALPZM000000000) [62]. RNA-seq data corresponding to the same strain (SRA accession: SRR23447917) were used for transcriptional evidence during the gene annotation. Predicted genes were further functionally annotated using emapper with EggNOG DB for consistency with the metagenomic data. Detailed methods for annotating genes from a pure genome are available in Method S5. After the gene prediction and annotation, we mapped the publicly available transcriptomic reads acquired from a pure isolate (designated as mycelia; in vitro expression), which were identical with those used in the gene annotation, and our metatranscriptomic reads (designated as TmD1 and TmM1; expression in soil conditions) to the annotated gene sequences of *Tm*’s whole genome using bwa, respectively. From these alignment data, mapped read counts of each gene were calculated and normalized to TPM using salmon.

### Viral metagenome-assembled genome detection and clustering

Tentative phages were recovered from the metagenomic contigs using virsorter2 (v. 2.0.0) [63] with the option *–include-groups dsDNAphage,NCLDV,ssDNA,lavidaviridae*. After sorting putative phage genomes, the quality of the sorted genomes was evaluated using CheckV (v. 1.0.1) [64]. Based on the CheckV results, qualified phage genomes, which size and completeness were over or equal to 5 kbp and 50%, respectively, were retrieved from the initially sorted genomes. The qualified phage genomes were further clustered to obtain representative phage genomes based on 95% average nucleotide identity and 85% alignment fraction following the MIUViG (Minimum Information about an Uncultivated Virus Genome) [65]. Taxonomy information of the representative phages was obtained using GeNomad (v. 1.7.4) [66] with default options. Based on the GeNomad results, we additionally excluded phage genomes classified as a plasmid. Additionally, we excluded the taxonomically unclassified phage genomes which belonged to *Tricholoma matsutake* after the blastn search.

### Viral gene annotation and quantification

From the detected phage genomes, we predicted functional genes using prodigal-gv (v. 2.11.0) [67]. Genes were annotated with default parameters. Then, functional annotation was conducted using mmseqs2 and the Prokaryotic Virus Remote Homologous Groups database (PHROGs v. 4) [68] with default parameters. To find the best hits from the initial annotation results, we screened them with the criteria: (i) alignment fraction is over or equal to 70%, (ii) *e* values are lower than 1e-05, and (iii) sequence identity is over or equal to 30%. After the first filtering, genes showing the lowest e-value were chosen in the second-round filtering.

To define auxiliary metabolic genes (AMGs) from the annotated genes, we first determined putative AMGs commonly found between the gene annotation results using PHROGs DB and DRAM-v (v. 1.5.0; the viral mode of DRAM) [69]. As a result, 600 putative AMGs were recovered. The genes were further annotated using KofamKOALA (v. 2025–02-01) [70], emapper with EggNog DB, COG (v. 2024) [71], and UniRef90 (accessed on March 8th, 2025) with mmseqs2. Initial annotation results were filtered with the *e* value cutoff 1e-05. To obtain consensus annotation results, each filtered annotation result was manually curated. Based on the curated annotation results, genes involved in “nucleotide metabolism” and “replication, recombination and repair” were excluded to remove illegitimate genes from the tentative AMGs. A total of 481 AMGs were determined from the identified phage genes. The expression levels of the predicted genes were quantified by mapping metatranscriptomic reads onto the DNA sequences of each gene using bwa. Further quantification was performed using salmon with the option *-l ISR* since the metatranscriptomic reads were obtained using a reverse strand-specific manner. The mapped reads were normalized to RPKM and TPM, respectively.

### Prediction of prokaryotic hosts and phage life strategies

Prokaryotic hosts of the identified phages were predicted using the Prokaryotic virus Host Predictor (PHP; https://github.com/congyulu-bioinfo/PHP) [72]. If the multiple prokaryotic hosts were assigned to a phage genome, the host information that score the highest was chosen. The life strategies of phages were predicted using PhaTYP (v. 0.3.0) [73] implemented in PhaBOX (v. 2.0) [74] web server (https://phage.ee.cityu.edu.hk/). We also manually predicted phages’ life strategies by manually detecting lysogeny-specific genes, including integrase, recombinase, transposase, excisionase, CI/Cro repressor, and parAB, as suggested in the previous study [75]. Consent phage life strategies were manually curated based on both results.

### Statistical analyses of the soil phage community data

The abundance of representative phages was estimated using CoverM (v. 0.7.0) [76] with metagenomic reads. The relative abundance of representative phages was determined by dividing read counts mapped to each phage genome by total read counts mapped to all phage genomes in each metagenomic sample. Beta diversity of the soil phage community was evaluated using identical approaches for the bacterial and fungal communities. Abundance-based hierarchical clustering of representative phages was performed using ComplexHeatmap (v. 2.18.0) [77]. To assess the relationships between representative phages and predicted prokaryotic hosts, linear regression analyses between phage and prokaryotic host abundances were performed with the function *lm* implemented in the R package stats (v. 4.3.3) [36]. *Tm* colonization pattern-associated AMG expression patterns were assessed using hierarchical clustering of gene expression patterns with ComplexHeatmap.

## Results

### Bacterial and fungal community properties in* T*. *matsutake *habitat

To examine the effect of *Tm* dominance on soil environments, we first investigated the composition of soil bacterial and fungal communities from *Tm*-minor (TmM) and dominant (TmD) soils. We initially obtained a total of 4,471,204 16S rRNA amplicon reads and 5,835,536 ITS1 amplicon reads from 50 soil samples. After quality control, a total of 7619 putative prokaryotic ASVs (1,413,380 reads) and 2082 putative eukaryotic ASVs (2,986,185 reads) were obtained. Of the prokaryotic reads, 1,412,990 reads were taxonomically classified, and 390 reads (seven ASVs) remained unclassified at the domain level. Of the eukaryotic reads, 2,981,269 reads were classified, and 4916 reads (106 ASVs) remained unclassified at the same taxonomic level. The final dataset consisted of 7502 bacterial ASVs (TmM: 4370 ASVs; TmD: 3942 ASVs) with 1,402,081 reads and 1944 fungal ASVs (TmM: 1620 ASVs; TmD: 834 ASVs) with 2,980,315 reads after removing undesirable taxa, including Archaea, chloroplasts, mitochondria (for bacteria), Viridiplantae, Rhizaria, Protista (for fungi), and domain-level unclassified ASVs. When the taxonomic distribution of the bacterial and fungal communities was examined, the compositional differences between TmD and TmM soils were observed across all sampling times (Figs. S2 and S3). For example, Actinomycetota (two-sided Welch’s *t* test [hereafter *t* test]: March, *P* = 0.0264; May, *P* = 0.0123; July, *P* = 0.0026; September, *P* = 0.0040; November, *P* = 0.0102) dominated the bacterial communities in TmD soil, whereas Acidobacteriota showed lower abundances compared to TmM soil (*t* test: March, *P* = 0.0113; May, *P* = 0.0380; July, *P* = 0.0026; September, *P* = 0.0017; November, *P* = 0.0040) from March to November 2022 (Fig. S2a; Table S1). Meanwhile, significant differences in the dominant fungal phyla, Ascomycota (*t* test: March, *P* = 0.3823; May, *P* = 0.3190; July, *P* = 0.5066; September, *P* = 0.0875; November, *P* = 0.0064) and Basidiomycota (*t* test: March, *P* = 0.3846; May, *P* = 0.6548; July, *P* = 0.7103; September, *P* = 0.6055; November, *P* = 0.2383) were not observed, except for Ascomycota in November (Fig. S3a; Table S1). However, the fungal compositions at the genus level showed a compositional discrepancy between TmD and TmM soils, though not statistically significant (Result S1; Fig. S3b; Table S1).

**Fig. 1:**
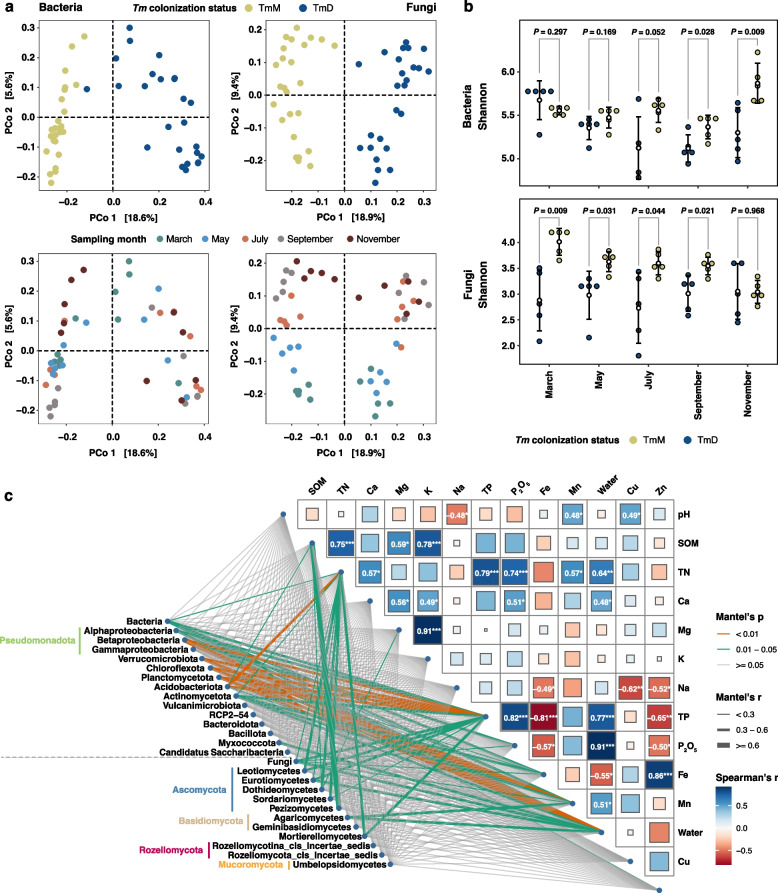
Properties of soil bacterial and fungal communities with *Tricholoma matsutake* (*Tm*) dominance. **a** Compositional variations in the bacterial (left panel) and fungal (right panel) communities depending on *Tm* dominance (upper panel) and sampling month (bottom panel). Ordination analysis was conducted by the principal coordinate analysis using cumulative-sum scaling normalized and log-transformed ASV abundances. **b** Shannon diversity values for the bacterial and fungal communities in each month. Dots correspond to the biological replicates of the TmD and TmM groups. White dots indicate the mean diversity values. Error bars show standard deviation values for each condition. Statistical significance was determined using Welch’s *t*-test after the normality test with the Shapiro–Wilk test. The exact data for the Shannon diversity values and statistical tests are available in Table S2. **c** Mantel test between the compositional distance of bacterial and fungal communities and environmental distance values. A heat map on the right side shows the correlation between chemical properties. The correlation coefficient values, which *p*-values are lower than 0.05, are depicted on the heat map tiles. The tile colors demonstrate correlation coefficient values (blue for positive correlations; red for negative correlations). The Mantel test was performed at the domain and phylum or class level. Dominant phyla and classes in Figs. S1 and S2 were chosen for the analysis. The significant correlations are indicated as green (correlations which *p* values are higher than 0.01 and lower than 0.05) or orange (correlations which p-values are lower than 0.01) lines. Non-significant correlations are indicated as gray lines. The thickness of the lines proportionally increases as Mantel’ *r* values increase

Ordination analysis further supported *Tm* condition-associated compositional variations in the bacterial and fungal compositions (Fig. 1a). The differences in the *Tm* conditions significantly contributed to the observed compositional variance in both bacterial and fungal communities (Table S2; two-way PERMANOVA, bacteria: R^2^ = 0.1811, *P* = 0.0001; fungi: R^2^ = 0.1740, *P* = 0.0001). Compared to the bacteria (R^2^ = 0.0980, *P* = 0.0054), the fungal community was more affected by the temporal factor, sampling month (R^2^ = 0.1744, *P* = 0.0001). Moreover, the interaction between *Tm* dominance and sampling month was also significant for both bacteria (R^2^ = 0.0943, *P* = 0.0086) and fungi (R^2^ = 0.1533, *P* = 0.0001) (Table S2), indicating that the effect of *Tm* dominance might vary depending on the sampling month. The dominance of *Tm* also led to the diminishment in bacterial and fungal alpha diversity (Fig. 1b; Table S2). This pattern was obvious in the fungal communities except in November. These results suggest that *Tm* dominance could alter the soil microbiomes irrespective of temporal effects.

To identify factors involved in the alteration of soil bacterial and fungal communities, we examined whether soil chemical factors are altered by the *Tm* colonization. A significant decrease in TN, TP, P_2_O_5_, Mn, and soil water contents, irrespective of the seasonal change, was found in the TmD soils compared to TmM soils (Result S2; Fig. S4; Table S3). Based on this chemical information, we hypothesized that *Tm*-associated changes in chemical environments could affect the compositional turnover of soil bacterial and fungal communities. Mantel test showed water content and TP levels commonly significantly affected the bacterial and fungal composition (Fig. 1c; Table S3). We additionally examined the effects of edaphic factors on microbial compositions at the phylum or class level. The assessment revealed differential influences of edaphic factors on microbial taxonomic groups. In the bacterial community, the water contents were a primary shaping factor for microbes in the dominant phyla, such as Actinomycetota and Chloroflexota, or classes, including Alphaproteobacteria, except for Acidobacteriota, for which TN was the most significant factor (Fig. 1c; Table S3). Actinomycetota and Chloroflexota explicitly showed a significant correlation with Fe contents. In the fungal community, Agaricomycetes was explicitly correlated with Mn contents but not with TP and TN, with which other dominant fungal classes were significantly correlated (Fig. 1c; Table S3). These results suggest the differential responses of resident microbes to *Tm*-associated environmental changes.

### *T. matsutake*-associated changes in the microbial functionality

Since we found remarkable shifts in the soil bacterial and fungal community compositions, we next assessed what functions could be changed along with the observed taxonomic alteration. Among the soil samples, we concentrated on soils collected in September when the fruiting bodies of *Tm* emerged. From the metagenomic data (Result S3; Table S4), the *Tm*-associated alteration of bacterial and fungal communities was also found (Fig. S5). Furthermore, the successional change of the *Tm* population across the years was identified (Fig. S5). In particular, hierarchical clustering and ordination analyses showed that the fungal metagenomic data were clustered into three main clusters, which were consistent with the *Tm* colonization history (Fig. S6). According to these *Tm* distribution patterns and clustering results, we further divided the soil sites into three groups based on the extent of *Tm* mycelial colonization in soil (as defined in Methods): pre-, active-, and post-colonization groups. When assessing the functional diversity of soil microbiomes, the ordination analyses at the gene level showed that soil bacteria (R^2^ = 0.5538, *P* = 0.0001) and fungi (R^2^ = 0.7012, *P* = 0.0001) were functionally differentiated by *Tm* colonization status (Fig. S7; Table S4).

**Fig. 2:**
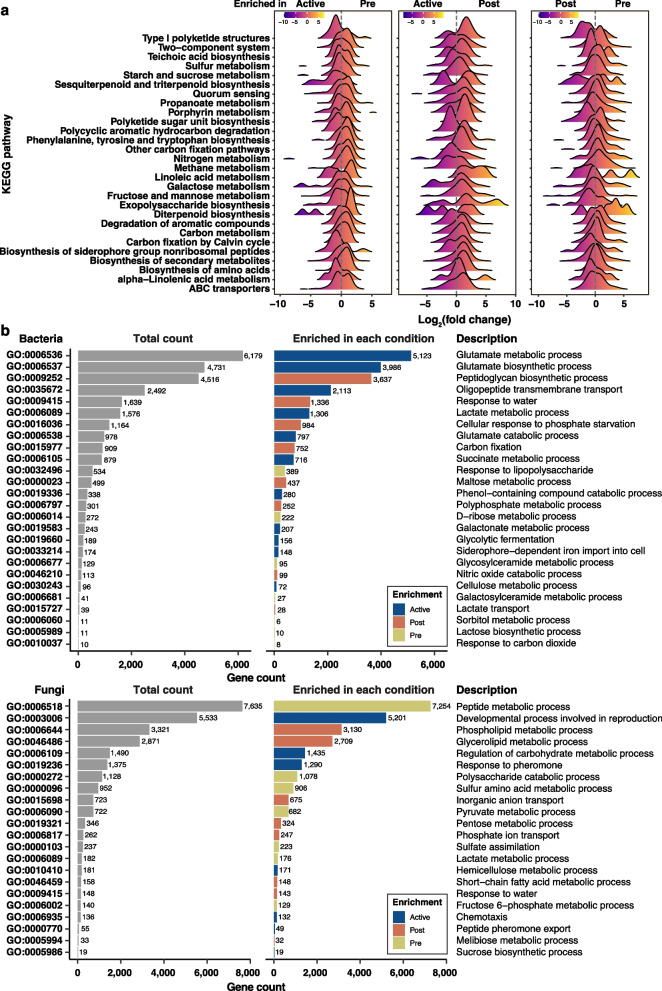
Functional differences of bacteriomes and mycobiomes by *Tricholoma matsutake* colonization.** a** The distribution of significantly abundant KEGG orthologs (KOs) in each pairwise comparison pair at the KEGG pathway categories. The colors of the density plots indicate the log_2_ fold change values of KOs in each KEGG pathway category. **b** Differentially abundant GO terms belonging to biological process and their gene counts. Bar colors indicate the group where GO terms belonged. Functional differences were investigated at the KO and GO term levels using DESeq2. The abundances of KOs and GO terms were estimated from the actual read counts mapped to the representative genes. Differential abundance assessment was performed in three pairwise comparison pairs (Pair 1, Active vs. Pre; Pair 2, Active vs. Post; Pair 3, Pre vs. Post) using DESeq2. Based on the differential abundance results, commonly abundant KOs or GO terms in the Pre, Active, and Post groups were used for the plots. Data regarding the differential abundant assessment are available in Table S5 and S6

To dig into differences in functional potentials by *Tm* colonization status, we further compared the abundance of Kyoto Encyclopedia of Genes and Genomes (KEGG) orthologs (KOs) and Gene Ontology (GO) terms using a differential abundance assessment. To find differential distribution of KOs in three *Tm* conditions, we examined the enrichment patterns of them in three pairwise comparison pairs (Pair 1, Active vs. Pre; Pair 2, Active vs. Post; Pair 3, Pre vs. Post) (Fig. 2a; Table S5). When differentially abundant KOs were grouped into each KEGG pathway categories, the shifts in functional enrichment patterns across pre-, active-, and post-colonization were found (Fig. 2a; Table S5). For instance, the numbers of enriched KOs belonging to type I polyketide structures, starch and sucrose metabolism, biosynthesis of siderophore group nonribosomal peptides, ABC transporters, and cell wall-related metabolisms (biosynthesis of exopolysaccharide and teichoic acid) increased in order Pre, Active, and Post groups. Those in two-component system, nitrogen metabolism, and methane metabolism showed the opposite patterns. The functional differences were also found at the GO term level. In the bacteriome, glutamate metabolism (GO:0006537 and GO:0006538), oligopeptide transmembrane transport (GO:0035672), and siderophore-related GO term (GO:0033214) were more abundant in the active colonization site (Fig. 2b; Fig. S8; Table S6). Meanwhile, GO terms related to response to lipopolysaccharide (GO:0032496) and peptidoglycan biosynthesis (GO:0009252) were abundantly distributed in pre- and post-colonization sites, respectively (Fig. 2b; Fig. S8; Table S6). The discrepancy in the functional potential was also detected in the mycobiomes. In the Active group, GO terms related to reproduction (GO:0003006, GO:0019236, and GO:0000770) were significantly abundant compared to other groups (Fig. 2b; Fig. S8; Table S6). These results indicated that *Tm* colonization accompanied the shift in chemical environments and compositional and functional properties of resident soil microbiomes.

### *T*.* matsutake*-associated metabolic shifting at the community level

**Fig. 3:**
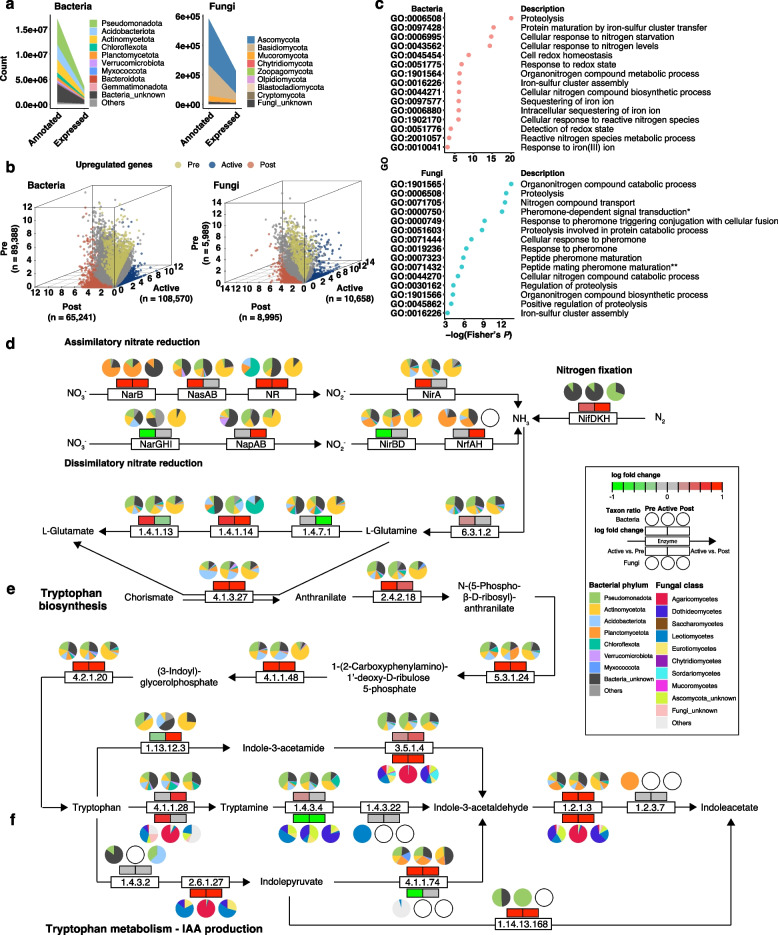
Metabolic landscape of bacterial and fungal communities according to *T*.* matsutake* colonization. **a** Annotated and expressed genes of the bacterial (left panel) and fungal (right panel) communities. **b** Specifically upregulated genes in each *Tm* colonization status group. Differentially expressed genes were predicted using the R package NOIseq. The values of the x, y, and z axes are log_2_ fold change values. Dots correspond to the expressed genes of bacteria (left panel) and fungi (right panel). Dots’ colors indicate the statistically significant upregulated genes in each group (yellow, Pre; blue, Active; red, Post). **c** GO term overrepresentation assessment of specifically expressed genes. GO overrepresentation assessment was conducted using the R package topGO. The significantly overrepresented GO terms related to nitrogen metabolism and reproduction were chosen. * Pheromone-dependent signal transduction involved in conjugation with cellular fusion. ** Peptide mating pheromone maturation involved in positive regulation of conjugation with cellular fusion. The original results are available in Table S10. **d**–**f** Pairwise comparison of gene expression levels at the KEGG pathway context. **d** Nitrogen reduction, nitrogen fixation, and glutamate biosynthesis. **e** Tryptophan biosynthesis. **f** Tryptophan-dependent indole-3-acetic acid biosynthesis. The colors of the boxes above (bacteria) or below (fungi) enzyme names or EC numbers indicate log fold change values in each comparison pair (Active vs. Pre and Active vs. Post; red, upregulated in the Active group; green, upregulated in the Pre or Post group; gray, non-differential regulation). The pie charts above (bacteria) or below (fungi) the expression boxes show taxonomic information of bacteria and fungi expressing corresponding metabolic genes. The original results are available in Figs. S10 and S11

Since the metagenomic data contain the functional potentials of dormant microbes, we further examined metabolically active functions from metatranscriptomic data. Among the representative genes, a total of 3,495,455 bacterial (20.53% of the annotated bacterial genes) and 226,531 fungal (39.1% of the annotated fungal genes) genes were actively expressed (Fig. 3a). We first examined specifically upregulated genes in each *Tm* condition. Pairwise differentially expressed gene assessments revealed that a total of 108,570 bacterial and 10,658 fungal genes were specifically upregulated in the Active-colonization group. Meanwhile, 95,377 and 74,236 genes were specifically upregulated in the Pre- and Post-colonization groups, respectively (Fig. 3b; Table S7 and S8). When sorting out the upregulated genes by their taxonomic affiliations, we found the differential contribution of microbial taxa to the upregulated genes. In the Active group, the bacterial genes were mainly possessed by Pseudomonadota (34.72%; unknown genus belonging to Alphaproteobacteria, 14.3%; *Bradyrhizobium*, 3.7%) and Actinomycetota (13.2%; unknown genus belonging to Actinomycetes, 3.7%; *Trebonia*, 1.0%), whereas the fungal genes were mainly affiliated to Agaricomycetes (*Tricholoma*, 81.1%) (Fig. S9; Table S9). Meanwhile, in the Pre and Post groups, upregulated bacterial genes belonged to Acidobacteriota and Actinomycetota, respectively. Fungal genes belonging to Dothideomycetes and Saccharomycetes were significantly upregulated in the Pre and Post groups, respectively. A GO term overrepresentation analysis with the identified upregulated genes revealed that GO terms related to reactive oxygen species (GO:0072593 and GO:0000302) and redox state (GO:0045454 and GO:0051775) were significantly enriched in the bacterial community in the Active group (Fig. 3c; Table S10). Meanwhile, reproduction-related terms (GO:0000750, GO:0000749, GO:0071444, GO:0019236, GO:0007323, and GO:0071432) were significantly overrepresented in the fungal community of the Active group, reflecting the reproductive status of *T*. *matsutake* (Fig. 3c; Table S10). In the bacterial and fungal communities, GO terms related to proteolysis and organonitrogen compound metabolisms were commonly enriched in the Active group (Fig. 3c; Table S10). The overrepresentation test at the KEGG pathway also showed the enrichment of genes involved in the metabolisms related to nitrogen (ko00910), glutamate (ko00250), and tryptophan (ko00380) in the Active group (Table S10).

Based on these results, we further examined nitrogen-related metabolisms. The pairwise comparison (Pre vs. Active and Active vs. Post) of KEGG ortholog expression levels showed the increased expression of the enzymes involved in assimilatory nitrate reduction (Fig. 3d; Figs. S10 and S12), tryptophan biosynthesis via shikimate pathway (Fig. 3e; Figs. S11 and S12), and tryptophan metabolism including indole-3-acetic acid (IAA) biosynthesis (Fig. 3f; Figs. S11 and S12) in the bacterial community. Microbes contributing to these metabolisms were shifted depending on the *Tm* colonization status. In the Pre group, most of the genes were expressed by bacteria belonging to Pseudomonadota, Acidobacteriota, and Planctomycetota, whereas the Post group was metabolically centered to Actinomycetota (Fig. 3d–f; Table S11). Meanwhile, the gene expression of the Active group was mainly governed by the microbes belonging to Pseudomonadota and Actinomycetota, suggesting the transition of metabolically active microbes by *Tm* colonization. Meanwhile, the identical enzymes were down-expressed in the fungal community of the Active group compared to the Pre and Post groups (Figs. S10 and S11), except for the IAA biosynthesis enzymes expressed by *Tm* (EC 4.1.1.28 tryptophan decarboxylase, EC 1.2.1.3 aldehyde dehydrogenase (NAD^+^), and EC 3.5.1.4 amidase) (Fig. 3f). These results suggest that *Tm* might lead to increased nitrogen demands in the bacterial and fungal communities.

### Microbial associations between *T. matsutake* and surrounding microbes

**Fig. 4:**
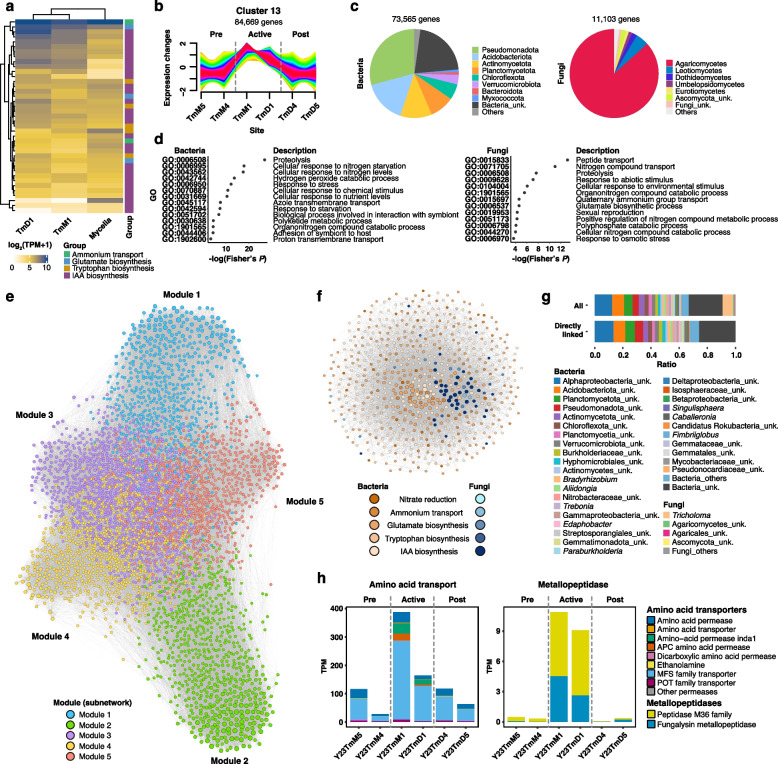
Metabolic associations between *T*. *matsutake* and surrounding microbial communities. **a** Comparison of the expression of the genes involved in ammonium transport, glutamate biosynthesis, tryptophan biosynthesis, and IAA biosynthesis between vegetative and reproductive stages of *Tm*. **b** Clustering of gene expression patterns. The original result of the gene expression pattern clustering is available in Fig. S12. **c** Taxonomic composition of bacteria and fungi in Cluster 13. **d** Overrepresentation test of the genes grouped into Cluster 13. **e** Gene co-expression patterns at the metatranscriptome level. Node colors indicate modules (or subnetworks) where each node belongs (blue, Module 1; green, Module 2; purple, Module 3; yellow, Module 4; red, Module 5). Gray lines show significant co-expression linkages between metagenomic genes. **f** Gene co-expression pattern of Module 1. Node colors represent functional groups (nitrate reduction, ammonium transport, glutamate biosynthesis, tryptophan biosynthesis, and indole-3-acetic acid [IAA] biosynthesis) of each gene. **g** Taxonomic affiliation of the genes in Module 1 and those directly associated with *Tm* genes. Ratio was estimated by dividing gene counts in each genus by total numbers of genes in Module 1 (All) or total numbers of genes which were directly connected with *T*. *matsutake* genes (Directly linked). **h** Expression level of fungal amino acid transporters or permeases and extracellular metallopeptidases. The exact values for the expression levels of amino acid transporters and metallopeptidases at each fungal species are available in Table S16

Although we found that *Tm* possessed incomplete IAA biosynthesis capacity from the metagenomic and metatranscriptomic data, we examined this functional defect in the pure genome of *Tm* owing to the possibility that genes were not detected by sensitivity or annotation matters. From the whole genome information with transcriptional evidence, we verified that the genes, such as tryptophan 2-monooxygenase (EC 1.13.12.3), monoamine oxidase (EC 1.4.3.4), diamine oxidase (EC 1.4.3.22), and indole-3-pyruvate decarboxylase (EC 4.1.1.74), were not detected, consistent with their absence in the metagenomic data (Table S12). Furthermore, we hypothesized that nitrogen demands by *Tm* might increase due to the IAA production during the reproductive stage. To prove this assumption, we compared the expression levels of nitrogen metabolisms between the vegetative (in vitro culture condition) and reproductive phases (soil condition). Compared to the mycelia, the genes involved in sexual reproduction were upregulated in the soil samples (Table S12), suggesting that *Tm* collected in this study underwent a reproductive phase. When the expression of the genes involved in IAA biosynthesis was compared, genes annotated to ammonium transporter, glutamine synthetase (EC 6.3.1.2), anthranilate synthase component I (EC 4.1.3.27), tryptophan aminotransferase (EC 2.6.1.27), aldehyde dehydrogenase (NAD +) (EC 1.2.1.3), and amidase (EC 3.5.1.4) were upregulated compared to the mycelial conditions (Fig. 4a; Table S12). These results indicate that *Tm* might require helpers to support its nitrogen demands and production of IAA under nitrogen starvation conditions at the reproductive stage.

To find potential microbes associated with *Tm*, we first assessed which genes show similar gene expression patterns with *Tm* genes. A total of 16 expression clusters were identified based on expression patterns by sites (Fig. S13). Among them, Cluster 13 consisted of 84,669 genes, including 8027 *Tm* genes (Fig. 4b), that mainly belonged to Pseudomonadata (*Bradyrhizobium*, 2086 genes; Burkholderiaceae, 798 genes), Acidobacteriota (*Edaphobacter*, 276 genes), and Actinomycetota (*Trebonia*, 581 genes; *Mycobacterium*, 448 genes) for bacteria and Leotiomycetes (*Oidiodendron*, 105 genes) for fungi (Fig. 4c; Table S13). Functions involved in the metabolism of nitrogen-related compounds were overrepresented in the bacteria and fungi, as shown in the genes highly expressed in the Active group (Fig. 4d; Table S13). In particular, the genes involved in nitrate reduction, glutamine biosynthesis, tryptophan biosynthesis, and IAA biosynthesis were clustered together (Table S13), suggesting metabolic associations around IAA between soil microbes and *Tm*.

To find potential metabolic associations between *Tm* and other microbes in the context of IAA biosynthesis, we constructed a metatranscriptomic gene co-expression network consisting of the genes involved in nitrogen metabolism and IAA biosynthesis. The resulting network contained 4048 nodes (genes) and 64,516 edges (associations) (Fig. 4e; Fig. S14; Table S14). The network was further divided into modules to identify a module with the highest number of *Tm* genes compared to the others. Module 1, among five modules, was further examined since the highest number of *Tm*’s genes were detected in it (Table S14), consisting of 615 genes with 9181 edges (Fig. 4f). In this module, unknown genera in Alphaproteobacteria, Acidobacteriota, Planctomycetota, Actinomycetota, and Chloroflexota were dominantly distributed. Among these taxa, *Conexibacter*, *Paraburkholderia*, *Bradyrhizobium*, and *Trebonia* were directly linked with *Tm* genes (Fig. 4g; Table S14). The possibility of *Conexibacter* and *Paraburkholderia* as putative *Tm* associates were also supported by microbial association network constructed with 2022 metabarcoding samples (Result S4; Fig. S15; Table S15). These results suggest that *Tm* might recruit specific bacterial helpers consisting of *Conexibacter* and *Paraburkholderia* having capabilities for metabolizing nitrogen substrates under nutrient-limiting conditions.

Based on this result, we assumed that *Tm* might actively express amino acid transporters or permeases to intake amino acids, in particular glutamate and tryptophan, from external environments. To assess this assumption, we examined the expression levels of fungal amino acid permeases or transporters genes involved in the import of amino acids. Among the permeases, genes encoding amino acid-polyamine-organocation (APC) amino acid permease and Major Facilitator Superfamily (MFS) known to be involved in the uptake of glutamate and tryptophan were highly expressed by *Tm* in the Active group (Fig. 4h; Table S16). Additionally, metatranscriptomic data showed that *Tm* also actively expressed Zn-dependent extracellular metallopeptidase genes (Fig. 4h; Table S16), which could be linked with the increased expression of amino acid permease or transporter genes. These results suggest that *Tm* might pursue two-track strategies, metabolic associations with helpers and scavenging of resources from environments, to meet its nitrogen demands under nitrogen-limiting conditions.

### *T*.* matsutake*-associated soil phage community

**Fig. 5:**
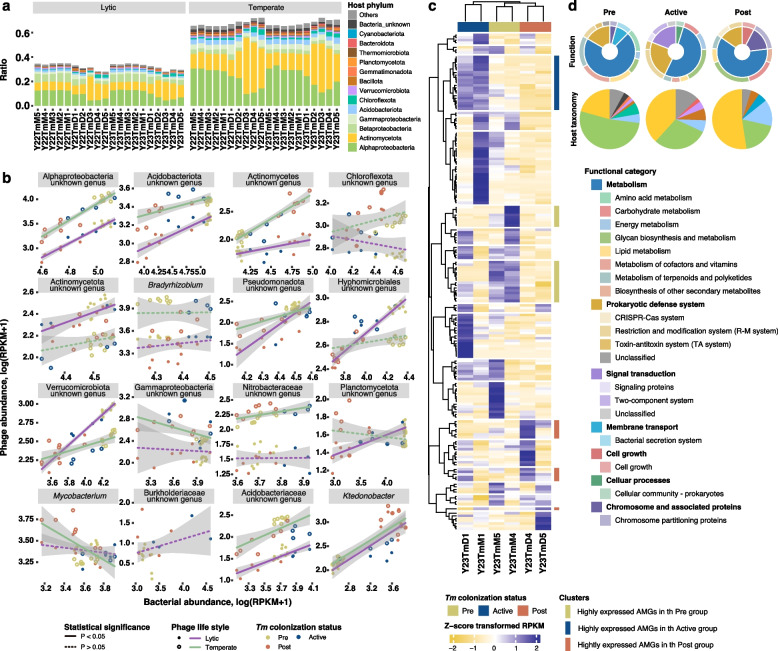
Host-associated alteration in soil phage communities depending on the *T. matsutake* colonization status. **a** Predicted host taxonomy of the soil phage communities and its composition. Each tick corresponded to the examined metagenomic samples. **b** Quantitative relationship between phages’ abundance and host bacterial abundance. The quantitative relationship between the phages and bacterial hosts was assessed based on the linear regression test. The abundances of the phages and bacterial hosts were determined based on metagenomic reads using CoverM (v. 0.7.0). The read counts were further normalized to reads per kilobase of transcript per million mapped reads (RPKM) values. The shape of dots indicates the phage lifestyles (filled circle, putative lytic phages; hollow circle, putative temperate phages). The lines show trend lines estimated using linear regression analyses (purple, trend line of putative lytic phages; green, trend line of putative temperate phages). The line shapes are designated by the statistical significance of the regression results (solid, *P* < 0.05; dashed, *P* > 0.05). The gray-shaded area indicates the 95% confidence area of each regression model. **c** Expression patterns of the predicted auxiliary metabolic genes (AMGs) across samples. The heat map was built based on the z-score transformed RPKM values using the R package ComplexHeatmap (v. 2.18.0). Rows and columns were clustered using Pearson’s correlation distance with the complete method. The colored boxes above the heat map indicate the sample groups designated by the colonization status of *T*. *matsutake* (*Tm*) (yellow, Pre; blue, Active; red, Post). The colored bars on the right of the heat map show the clusters where genes are highly expressed in each condition (yellow, Pre; blue, Active; red, Post). **d** Functional composition of the AMGs in the chosen clusters and host taxonomic composition of the phages carrying the corresponding AMGs. The colors of each phylum are identical to those in Fig. 5a, except for the green section indicating Pseudomonadota

Since we found the *Tm*-associated alteration in the bacterial community structure and functions, we additionally examined the shift in the parasite community against bacteria (bacteriophages) and its effects on the host community. From the metagenomic and metatranscriptomic samples, we revealed 3246 representative DNA phages consisting of 1291 lytic and 1955 temperate phages (Result S5; Figs. S16 and S17; Table S17). Most of the identified phages were classified as Caudoviricetes where bacteriophages belonged (Result S5; Fig. S17a). The soil phageome was also clustered separately along with the *Tm* colonization status as presented in the bacterial community (Result S3; Fig. S17b). Based on this finding, we next assessed whether the composition of potential bacterial hosts was similar to the previously observed bacterial community composition. In both lytic and temperate phages, similar to the bacterial community composition, phages that possibly infect Actinomycetota were dominant in the Post group, whereas those able to infect Acidobacteriota and Pseudomonadota were highly abundant in the Pre and Active groups (Fig. 5a; Table S17). We additionally performed regression analyses between the dominant phages and bacterial host abundances, revealing significant positive correlations between the dominant lytic and temperate phages and corresponding host taxa, except for *Mycobacterium* showing significant negative correlations (Fig. 5b; Table S17). From the metatranscriptome data, we further examined active phages expressing lysis-related genes (hereafter active lytic phages), including holin, lysozyme, and endolysin. We found that the ratio of the active lytic phages gradually decreased from Pre to Post groups (Fig. S18a). Irrespective of the decrease in the active lytic phages, a total of 85 phages predicted to actively express lysis-related genes and infect dominant bacteria in each sample were detected (Fig. S18b; Table S17). These results suggest that kill-the-winner and piggyback-the-winner strategies coexisted in phage-bacteria interactions in the examined samples.

Since phages could harbor additional genes called auxiliary metabolic genes (AMGs) to support hosts’ physiology for their proliferation, we aimed to examine the presence of AMGs and their expression. We found 481 AMGs involved in the various metabolisms such as carbohydrates, amino acids, and lipids; signal transduction; and cell growth regulation (Table S17). To determine whether the expression patterns of AMGs are associated with the *Tm* colonization status, we performed hierarchical clustering of phages’ AMGs using the metatranscriptome data. Through the analysis, we found that the expression patterns of AMGs differed along with the *Tm* colonization status (Fig. 5c). Based on their expression patterns, the AMGs were additionally grouped into 20 clusters. Among them, six gene clusters showed distinct expression patterns depending on the *Tm* colonization status (Table S17). When investigating the functional properties of each cluster, we found that functions involved in glycan biosynthesis and metabolism, such as exopolysaccharide (K16555 and K16568) and teichoic acid biosynthesis (K19002), were enriched in the Active and Post groups compared to the Pre group. Meanwhile, those involved in the biosynthesis of antibiotics (K15669, K16381, and K16437) and amino acid metabolism (K00472, K00640, and K00797) were more expressed in the Pre group (Fig. 5d; Table S17). The host taxonomy of the phages expressing the observed distinct AMGs corroborated the shifts in the phage community linked with the bacterial host community (Fig. 5d). These results suggest that the phage community might contribute to the shift in the bacterial community by providing additional metabolic capabilities to adapt environmental changes associated with *Tm*.

## Discussion

Multi-omics approach-based studies on ECM systems, such as the *Suillus pungens*-*Pinus muricata* D. Don (Bishop pine) system, have enlightened our understanding of the ECM fungi-associated microbiome-driven soil ecosystem dynamics and metabolite-wide multi-domain interactions in soil environments [78, 79]. However, the biology underlying *Tm* and the surrounding abiotic and biotic environments has been barely understood compared to other ECM fungi. Previous studies on *Tm* have mainly concentrated on the formation and biological activities of plant-mycorrhiza associations during the vegetative growth of the fungus and microbial contributors [13, 21, 27, 28]. In this study, we addressed this gap by uncovering *Tm*-associated changes in abiotic and biotic environments, including bacteria, fungi, and phages, using an integrative multi-omics-based strategy that enables high-resolution mapping of *Tm*-associated ecological restructuring.

We found *Tm*-associated changes in soil chemical properties, in particular, soil moisture, Mn, and P, and their influence on bacterial and fungal compositional turnover (Fig. 1; Fig. S4). *Tm* is known to actively absorb and accumulate trace elements, including Mn and P, in its hyphae from rock fragments during growth [80]. This active uptake of soil inorganic ions by *Tm* might be involved in the decrease in the concentration of soil inorganic matter. Mycorrhiza colonization, including *Tm*, also could lead to a decrease in soil water contents in the loam soil [81] and soils where the shiro is developed [82], suggesting that changes in soil structure by fungal hyphae and mycorrhiza-mediated active water uptake might involve the lowering of soil moisture. Using the parallel assessment of microbial composition and soil abiotic factors, we found that TN, TP, Mn, and soil moisture were significant factors in shaping the bacterial and fungal communities (Fig. 1c; Table S3). In particular, Alphaproteobacteria, Acidobacteriota, and Actinomycetota of the bacterial community and Leotiomycetes, Eurotiomycetes, and Dothiodeomycetes of the fungal community were significantly affected by the alteration of those abiotic factors. A previous study reported that soil nitrogen and phosphorus contents were significant environmental drivers shaping bacteria and fungi in the *Tm*-colonizing soil [27]. These results suggest that Tm colonization might foster the alteration of microhabitats where other microbes reside and induce community-wide compositional changes.

Metagenome data revealed the community-wide metabolic adaptation to *Tm*-induced environmental changes. We revealed that glutamate-related functions were enriched in the soils where *Tm* was actively colonized. The bacterial communities in the Active group abundantly possessed glutamate biosynthesis-related genes compared to other soil conditions (Fig. 2b). Glutamate and glutamine generally contribute to pH homeostasis, osmotic balance, and cellular protection against low pH and desiccation. For instance, desiccation-tolerant *Arthrobacter* and *Rhodococcus* spp. could accumulate glutamine and glutamate as part of stress exudates, alongside trehalose and β-hydroxybutyrate [83]. Additionally, functions associated with the bacterial cell wall, such as the peptidoglycan biosynthetic process, teichoic acid biosynthesis, exopolysaccharide biosynthesis, and response to water were enriched as environmental conditions became barren (Fig. 2a). Previous studies showed that microbial communities surrounding mycorrhiza are generally assembled by mycorrhiza-involved changes in microenvironments and nutrient availability. For example, arbuscular mycorrhizal fungi are known to release carbon-rich exudates consisting of glucose and trehalose to recruit specific microbial taxa such as phosphate-solubilizing bacteria [84, 85]. Similarly, ectomycorrhizal fungi also could secrete hyphal exudates, including antimicrobial substances like hydrogen peroxide, leading to selective enrichment of specific bacterial taxa [86–89]. *Tm* is also known to secrete hydrogen peroxide as a part of exudates [90]. Our findings that genes involved in the metabolism of reactive oxygen species were enriched in the Active group (Fig. 3c; Table S10) suggest that bacteria having the ability to endure *Tm*-associated abiotic arid stress and biotic oxidative stress might be selected during *Tm* colonization. Given the context dependency in plant-ECM fungus-microbe associations [91, 92], culture-dependent approaches will help identify the factors affecting the assembly of the soil microbial community surrounding *Tm*, which meta-omics-based approaches may miss.

Ectomycorrhizal fungi, which retain saprophytic ancestry [93], could secrete extracellular proteases like metallo-, serine-, and aspartic peptidases, allowing them to access organic nitrogen pools from proteins, peptides, and microbial necromass in the soil [94–97]. Our result that *Tm* could release M36 family metallopeptidases to obtain nitrogen sources also corroborates the previous findings, suggesting that *Tm* actively scavenges nitrogen sources via saprophytic activity under nitrogen-limiting conditions. We also found that organonitrogen metabolism-related functions, including peptide degradation and transport, and cellular response to nitrogen starvation were upregulated in the bacterial communities in the Active group (Fig. 3c) and showed co-expression patterns with the *Tm* genes (Fig. 4d). This co-expression of mycorrhizal and bacterial nitrogen metabolism-related genes suggests enhanced nitrogen mining under nitrogen-poor conditions. Previous studies on ECM fungi, such as the ECM community associated with *Fagus sylvatica*, also reported that enhanced nitrogen mining via enzyme secretion, degradation of organic nitrogen compounds, and nutritional competition and cooperation between ECM fungi and the surrounding bacteria in nitrogen-limiting forest or soil environments, ultimately affecting the nitrogen acquisition of host plants [78, 98–100]. Similarly, a previous study of an arbuscular mycorrhizal (AM) fungus *Rhizophagus irregularis* reported facilitated nitrogen acquisition of the plant *Brachypodium distachyon* by the interaction between the AM fungus and the nearby soil microbial community [101], although underlying mechanisms were not covered. Another study also reported that hyphosphere bacteria of *Morchella* spp. can enhance the activity of fungal proteolytic enzymes, leading to increased hydrolysis of organic nitrogen compounds, suggesting that bacteria not only produce their own proteolytic enzymes but also amplify fungal enzyme activity, thereby improving nitrogen availability in the ecosystem [102]. Recent conceptual advances also emphasize that the hyphosphere is a dynamic hotspot for microbial interactions and nutrient transformations, which reframes the traditional rhizosphere-centric view [103]. These findings suggest that mycorrhiza may develop a microbe-associated strategy, particularly specialized hyphosphere microbiomes, for exploiting environmental sources effectively and efficiently. In this context, *Tm* may also pursue a coordinated microbial strategy for efficient nitrogen mining and acquisition in a nitrogen-limited environment. Further studies on *Tm*-derived metabolites and signaling factors that involve metabolic synchronization with a nearby hyphosphere microbial community will shed light on the ecological understanding of *Tm*-soil microbe interactions.

Consistent with the previous report [104], we confirmed that *Tm* actively expressed IAA biosynthesis genes with incomplete pathways. In *T*. *vaccinum*, an ectomycorrhizal fungus phylogenetically close to *Tm*, IAA influences hyphal morphology and Hartig net formation on roots [19], suggesting the roles of IAA in fungal vegetative growth and symbiotic interactions with host plants. In our study, the expression of IAA biosynthesis genes was upregulated during the reproductive stage compared to in vitro conditions at the vegetative stage. Given this point, it could be inferred that IAA might have unidentified functions devoted to *Tm*’s reproduction. In *Lentinus tigrinus*, a wood-rotting fungus belonging to Polyporales in Agaricomycetes, IAA is involved in the multiplication of fruiting bodies and an increase in the fresh weight of them [105]. The compound is also known to contribute to the differentiation of fruiting bodies, particularly primordium formation, of *Volvariella volvacea*, a non-mycorrhizal fungus belonging to Agaricales in Agaricomycetes [106]. These reports suggest that IAA might be involved in *Tm*’s fruiting body formation as well as the mycorrhizal assembly on roots. We discovered the enrichment of IAA biosynthesis-related metabolic pathways in the *Tm*-associated bacterial community. In particular, metatranscriptome-based gene co-expression networks showed the metabolic linkage from nitrate reduction to IAA biosynthesis (Fig. 4e–g). We confirmed that bacterial taxa, such as *Bradyrhizobium* [17] and *Paraburkholderia* [107], known as mycorrhiza helper bacteria (MHB), participated in this metabolic connection. A recent study reported the dependency of MHB, such as *Burkholderia*, on the ECM fungi [108], suggesting that these bacterial activities may be dependent on the presence of *Tm*. Further experimental studies would provide insights into *Tm*-dependent metabolic capabilities of the identified bacteria and their effects on *Tm*.

Although our metatranscriptome-scale gene co-expression network analysis revealed potential functional connections among *Tm* and other microbial genes, it should be noted that SparCC-based network inference has inherent limitations. As this method assesses only pairwise correlations, it may identify spurious associations that arise from indirect relationships mediated by third variables rather than direct functional interactions [109]. While these limitations do not invalidate the overall patterns observed in our network analysis, the specific gene–gene associations should be interpreted cautiously. Future studies employing experimental validation, such as controlled co-culture experiments of *Tm* and other microbes, would help refine these association predictions.

Among the known MHB, the bacteria belonging to *Paenarthrobacter*,* Bacillus*, and *Klebsiella* are known to produce IAA, ultimately enhancing mycelial growth and mycorrhiza formation [110–112]. Considering that intact IAA biosynthesis pathways were recovered at the community level, *Tm*-associated bacterial community might provide metabolic abilities unidentified in *Tm*, such as decarboxylation of indole pyruvate to indole-3-acetaldehyde or oxidation of tryptamine to indole-3-acetaldehyde. *Conexibacter*, which has not been reported as MHB, was also revealed as a potential associate of *Tm*. Previous studies showed that *Conexibacter* spp. involves nitrogen transformations such as nitrate reduction [113] and ammonia assimilation [114], suggesting key roles in the nitrogen cycling under nitrogen-limiting conditions. Further investigation using nitrogen isotopes-labeled inorganic nitrogen and IAA precursors will help us to understand nitrogen-centered metabolic concert in the microbial community modulated by *Tm* and microbial helpers involving this association.

Another prominent finding is that *Tm* colonization affected not only the adjacent bacterial community but also its parasitic phage community. We revealed that kill-the-winner and piggyback-the-winner strategies coexisted in the phage-bacteria interactions (Fig. 5a, b). Previous studies reported the ecological impact of these two strategies on bacterial ecology and functioning. Specifically, active lytic phages might suppress dominant bacteria, potentially maintaining bacterial diversity [115]. Their lytic activity also possibly leads to a viral shunt, suggested as the “leaky goods hypothesis” [116], which could provide nutrients, including organic matter and diffusible metabolites, to ECM fungi and other nearby microbes by lysing host cells [117]. This possibility further suggests the active roles of phages in the resource dynamics and nutrient-driven microbial associations in the hyphosphere of ECM systems. Meanwhile, temperate phages might stabilize and protect bacterial populations, leading to the coexistence of the soil bacteria at low resource influx by minimizing phage predation pressure [118]. This coexistence suggests that phages might contribute to maintaining both short-term stability and long-term adaptability of the host community, ensuring their continued ecological success under harsh environments. Further temporal investigation of phage communities and signaling cues driven by environments and bacteria will foster the understanding of ecological mechanisms of the shift in phages’ life strategy and its effects on the bacterial community. Phage community-wide functional landscapes covered by AMGs also emphasized the possibility of the enhanced environmental adaptation of the soil bacteria (Fig. 5c, d). In particular, phages infecting *Bradyrhizobium* highly expressed a function related to exopolysaccharide production in the Active group where the genus was most abundant. Meanwhile, phages infecting the bacterial genera *Mycobacterium* and *Arthrobacter* belonging to Actinomycetota highly expressed the gene involved in the endospore formation compared to other soil conditions. Previous studies reported that AMGs encoded by phages could improve bacterial fitness under resource-limiting conditions. Cyanobacteria infected by cyanophage S-PM2d showed continuous CO_2_ fixation and delayed cell death during light-limiting conditions [119]. Phage-infected sulfur-oxidizing *Thiomicrospira* in the hydrothermal vent also showed enhanced thiosulfate oxidation ability compared to non-infected bacteria [120]. Similar to these cases, a recent study of plant-mycorrhiza-bacteria interactions proposed the “auxiliary recruitment hypothesis” [116], which states that lysogenic conversion of MHB by AMG-possessing phages promotes mycorrhizal symbioses. Although we could not confirm whether AMG-possessing phages infect potential MHB associated with *Tm* and enhance mycorrhizal associations, the possibility that the phages with AMG in this study may foster bacterial adaptation to *Tm*-induced environmental changes implies the broader ecological significance of phage-associated auxiliary metabolisms in *Tm*-mediated mycorrhizal systems. Further isolation and experimental assessments of the phages covered in our study will allow us to reveal facilitated adaptation of the bacteria under *Tm*-induced environmental fluctuations and ecological and biological impacts on the pine tree-*Tm*-microbiome continuum.

## Conclusions

Mycorrhiza-plant associations are widely understood in the binary interaction-centered conceptual framework. With the discovery of mycorrhiza helper bacteria, this conceptual framework has been expanded to ternary interactions. Our findings showed that mycorrhizal associations are more intricate biological and ecological systems than the previously discovered. Although our study did not include culture-dependent experimental approaches, which limited the assessment of context-dependent features in ECM systems, and did not cover geographic diversity, we revealed unique environmental changes by *Tm* compared to other mycorrhizal systems and metabolic synchronization between *Tm* and the surrounding bacterial community using the integrative meta-omics approaches. In addition, we proposed potential ecological roles of the soil phage community infecting the bacterial community in the *Tm* mycorrhizal system. Our study will provide ecological insights into the hidden biological and ecological properties in the soil microhabitats affected by *Tm*. Collectively, our findings have important implications for *Tm* cultivation. The identification of potential helper bacteria and their metabolic synchronization with *Tm*—particularly in nitrogen metabolism, glutamate biosynthesis, and IAA production—provides targets for developing beneficial microbial consortia. Our characterization of *Tm*-associated abiotic factors, including soil moisture, nitrogen, and phosphorus, offers practical guidelines for soil amendment and site selection. Additionally, the finding that phages potentially modulate bacterial fitness through AMGs suggests phage-based strategies for maintaining bacterial communities favorable for *Tm*’s mycelial colonization and fruiting body formation. Future culture-dependent experiments can leverage these insights through several approaches: co-inoculation with identified helper bacteria, targeted soil conditioning based on characterized abiotic factors, and phage-mediated community engineering. These microbiome-informed *Tm* cultivation strategies, when validated across multiple sites, will advance practical applications for *Tm* cultivation. Ultimately, such approaches will improve yield stability in *Tm* production systems.

## Supplementary Information

Additional file 1.

Additional file 2.

## Data Availability

Raw sequence data generated and analyzed in this study were deposited in the NCBI Sequence Read Archive (metabarcoding data, PRJNA1249464; shotgun metagenome data, PRJNA1249386; metatrascriptome data, PRJNA1249434). All codes used in this study are available at GitHub (https://github.com/hyunkim90/TmMicrobiomeProject).

## Abbreviations

APC: Amino acid-polyamine-organocation
ASV: Amplicon sequence variant
ECM: Ectomycorrhiza
GO: Gene Ontology
IAA: Indole-3-acetic acid
ITS: Internal transcribed spacer
KEGG: Kyoto Encyclopedia of Genes and Genomes
KO: KEGG ortholog
MFS: Major Facilitator Superfamily
MHB: Mycorrhiza helper bacteria
NMDS: Non-metric multidimensional scaling
PERMANOVA: Permutational multivariate analysis of variance
PNA: Peptide nucleic acid
rRNA: Ribosomal RNA
spp.: Species
*Tm*: *Tricholoma* *matsutake*
