# Supplementary material for: Multi-meta-omics reveal unique symbiotic synchronization between ectomycorrhizal fungus and soil microbiome in Tricholoma matsutake habitat
Source: Microbiome. 2025 Dec 11;14:23. doi: 10.1186/s40168-025-02292-7 (PMC12801662; doi:10.1186/s40168-025-02292-7)
Supplement: Supplementary file 2 — Additional file 1. [file 40168_2025_2292_MOESM1_ESM.pdf]

Supporting Information of

Multi-meta-omics reveal unique symbiotic synchronization between ectomycorrhizal fungus and soil microbiome in *Tricholoma matsutake* habitat

In Hyup Bae[1†], Hyun Kim[1,2†*], Su-Min Kim[1], and Yong-Hwan Lee[1,2,3,4*]

[1]Department of Agricultural Biotechnology, Seoul National University, Seoul 08826, Republic of Korea

[2]Research Institute of Agriculture and Life Sciences, Seoul National University, Seoul 08826, Republic of Korea

[3]Interdisciplinary Programs in Agricultural Genomics, Seoul National University, Seoul 08826, Republic of Korea

[4]Plant Immunity Research Center, Center for Plant Microbiome Research, and Plant Genomics and Breeding Institute, Seoul National University, Seoul 08826, Republic of Korea

[†]These authors equally contributed to this study.

*Correspondence

Yong-Hwan Lee (yonglee@snu.ac.kr)

Hyun Kim (kh0219@snu.ac.kr)

This file includes:

Methods S1-S6

Results S1-S5

Figs. S1-S18.

Method S1 Soil chemistry

A total of 300 g of each soil was used to analyze soil texture (contents of sand, silt, and clay) and chemical properties (pH, soil organic matters [SOM], total nitrogen [TN], total phosphorous [TP], exchangeable Ca^{2+}, Mg^{2+}, Na^{+}, K^{+}, Fe, Mn, Cu, Zn, phosphate [P_2O_5], and water content). To measure soil pH, 5 g of air-dried and sieved soils were added into 25 ml of deionized water and mixed for 30 min. The soil suspension was incubated for 1 h at room temperature. pH was measured using a pH meter (HM-30R, DKK-TOA, Japan). SOM and TN contents were measured following Walkley-Black methods[1] and Kjeldahl method [2], respectively. To quantify the contents of exchangeable cations, 5 g of air-dried soils were added into 50 ml of 1N NH_4OAc (pH 7.0). After incubation for 30 min, the contents of cations were measured using inductively coupled plasma (ICP) emission spectroscopy (ICP-730-ES, Varian, United States; ICP-7510, Shimadzu, Japan; ICP-7400, Thermo Fisher Scientific, United States). The contents of P_2O_5 were measured following the Bray No.1 method [3].

Prior to measuring the levels of soil metals, soil samples were preprocessed first. A wind-dried soil sample was sieved using a mesh (pore size: 0.15 mm). And then, 3 g of the sieved soil was transferred to a 250 ml reacting flask combined with a cold-trap reflux condenser. A total of 21 mL of HCl and 7 mL of HNO_3 were added to the soil and mixed completely. A total of 15 mL of 0.5 M HNO_3 was added to the mixture. The mixtures were incubated at room temperature for 2 h to oxidize soil organic matter. The oxidized mixtures were heated until reaching the reflux condition. After that, the mixtures were incubated for 2 h while maintaining the reflux condition. The supernatant of the mixture was filtered using a filter paper (Whatman No. 40, Maidstone, United Kingdom) until fitting the mark in a 100 ml volumetric flask. The levels of Fe, Cu, Mn, and Zn were measured using inductively coupled plasma optical emission spectroscopy (ICP-OES, Agilent, Santa Clara, United States). The levels of soil metals were quantified using the following mathematical formula.

$$\text{Soil metal content (mg/kg)} = \frac{C_1 - C_2}{W_d} \times f \times V$$

C_1: metal concentration of a sample (mg/L) estimated from a calibration curve

C_0: metal concentration of a reference solution estimated from a calibration curve

f: dilution factor (when is out of the range of a calibration curve)

V: the volume of a flask (here 0.1 L)

W_d: drying weight of a soil sample

Soil water contents were measured following the procedure described below.

A weighing bottle was cooled in a desiccator after being dried in a drying oven (CO-81; HYSC, Seoul, Republic of Korea) at 105 to 110 °C for 1 h. After that, the weight of the dried bottle was measured (W1, the weight of a bottle). And then, a soil sample was added to the bottle, and the weight of the bottle with the soil was measured (W2, the weight of both a bottle and soil sample before drying). Soils were incubated and dried in a drying oven at 105 to 110 °C for at least 4 h. The weight of the dried soil samples was measured after being cooled in a desiccator (W3, the weight of both a bottle and soil sample after drying). The resulting soil water contents were calculated using the following mathematical formula.

$$\text{Soil water content (\%)} = \frac{\text{W2} - \text{W3}}{\text{W2} - \text{W1}} \times 100$$

W1: the weight of an empty weighing bottle
W2: the total weight of a bottle and a soil sample before drying
W3: the total weight of a bottle and a soil sample after drying

All edaphic factors were analyzed in the National Instrumentation Center for Environmental Management (NICEM) at Seoul National University, Republic of Korea.

Method S2 Metabarcode generation

The V4 regions of bacterial 16S ribosomal RNA (16S rRNA) genes were amplified using universal 515F (5′-TCGTCGGCAGCGTCAGATGTGTATAAGAGACAGGT GCCAGCMGCCGCGGTAA-3′) and 806R (5′-GTCTCGTGGGCTCGGAGATGTGT ATAAGAGACAGGGACTACHVGGGTWTCTAAT-3′) PCR primers [4]. To reduce plant mitochondrial and plastid DNA contamination, peptide nucleic acid (PNA) PCR blockers (Panagene, Daejeon, Republic of Korea) (mPNA, 5'-GGCAAGTGTT CTTCGGA-3'; pPNA, 5'-GGCTCAACCCTGGACAG-3') were added during the first round PCR [5]. For fungi, the ITS1 region of the nuclear ribosomal RNA gene cluster was amplified using ITS1F (5'-TCGTCGGCAGCGTCAGATGTGTATAAGAGACAG CTTGGTCATTTAGAGGAAGTAA-3') and ITS2 (5'-GTCTCGTGGGCTCGGAGA TGTGTATAAGAGACAGGCTGCGTTCTTCATCGATGC-3') PCR primers [6] to increase the probability of acquiring fungi-specific reads. Each sample was amplified in triplicate. The PCR mixture consisted of 12.5 μL of Invitrogen Platinum SuperFi II Green PCR Master Mix (Thermo Fisher Scientific), 0.5 μM of each forward and reverse

primer, 0.8 μM of diluted DNA template, and pPNA and mPNA (0.75 μM each). For the ITS libraries, the conditions were identical, except that PNA clamps were not used. PCR was performed with initial denaturing at 98°C for 30 s, followed by 32 cycles of denaturing at 98°C for 10 s, PNA annealing at 78°C for 10 s, primmer annealing at 55°C for 10 s, and extension at 72°C for 60 s. The program was similar to ITS PCR amplification, but it lacked the PNA annealing step. Amplicon replicates were pooled and purified using the MEGAquick-spin™ Plus DNA Purification Kit (iNtRON Biotechnology, Seongnam, Republic of Korea) with an additional ethanol clean-up step to remove unused PCR reagents and resulting primer dimers. Second-round PCR was conducted using the Nextera XT Index Kit (Illumina, San Diego, United States). Each sample was amplified in a 50 μL reaction tube. The PCR mixture contained 25 μL of 2×KAPA HiFi HotStart ReadyMix (Roche, Basel, Switzerland), 5 μL of each forward (Nextera Index primer 1) and reverse primer (Nextera Index primer 2), and 100 ng of diluted DNA template. The final reaction volume was fitted by adding nuclease-free water. PCR was performed with initial denaturing at 95°C for 3 min, followed by 8 cycles of denaturing at 95°C for 30 s, primer annealing at 55°C for 30 s, and extension at 72°C for 30 s and final extension at 72°C for 5 min. DNA templates were diluted to equal concentrations based on measurements by the Infinite 200 pro (Tecan, Männedorf, Switzerland). The libraries were pooled and concentrated using AMPure beads (Beckman Coulter, Brea, United States). Pooled libraries were subjected to gel purification to remove unwanted PCR products and sequenced on the Illumina MiSeq platform with a 2 × 300 bp read length.

Method S3 Preparation of whole metagenomic samples

The genomic DNA was extracted from the soil samples collected in September 2022 (n = 10) and 2023 (n = 10) using SPINeasy® DNA Pro Kit for Soil (MP Biomedicals, CA, USA) following the manufacturer's instructions with a few modifications. The first modification was at the homogenization step, and the instruction recommended a single cycle of bead beating at 5 m/s for 35 s using FastPrep-24™ 5G bead beating system (MP Biomedicals, CA, USA), but the maximum yield was achieved at two cycles at 4m/s for 30s. Also, the elution volume of 100 μL was applied at the elution step to achieve maximum yield. Extracted genomic DNA was stored in a deep freezer at -80 °C until it was sent to the sequencing company for the quality control (QC).

The extracted genomic DNA samples were sent to Macrogen (Seoul, Republic of Korea) for the WGS sequencing. Agilent gDNA ScreenTape System or 2100

Bioanalyzer (Agilent Technologies, CA, USA) was used to check the quality, quantity, and size of DNA. The passed genomic DNA from the DNA QC was used to the library construction. The library was prepared with TruSeq Nano DNA Library Prep Kit (Illumina, CA, USA). The constructed library was screened with Agilent D1000 ScreenTape System (Agilent Technologies, CA, USA) to check the size and quantity of the constructed library. The quality-controlled libraries were sequenced with NovaSeq 6000 System (Illumina, CA, USA), and the raw data were generated in basis of 151 bp paired-end reads and 10 Gbp.

Method S4 Preparation of metatranscriptomic samples

For RNA extraction from the soil samples collected in September 2023 (n = 6; TmM5, TmM4, TmM1, TmD1, TmD4, and TmD5), the SPINeasy® DNA/RNA Kit for Soil (MP Biomedicals, Irvine, CA, USA) was utilized following the manufacturer's instructions, with three modifications to optimize the protocol for low-biomass soils. To minimize RNA degradation during the freeze-thaw cycle, soil samples stored at -75 °C were first transferred to 4 °C prior to being added to the lysing tube. After processing, any remaining soil samples were snap-frozen with liquid nitrogen to effectively preserve RNA integrity for potential future experiments. Additionally, the handling time of soil at room temperature was restricted to less than 5 minutes to further minimize the risk of RNA degradation. First, 500 mg of soil per sample was used in the initial lysing tube to maximize RNA yield. Second, 8 μL of β-mercaptoethanol (Sigma-Aldrich, St. Louis, MO, USA) was added to the tube prior to the homogenization step to enhance RNA stability and reduce degradation. Homogenization was performed using the FastPrep-24™ 5G bead-beating system (MP Biomedicals, Irvine, CA, USA) following the manufacturer's standard settings (35 sec at 5 m/s). Third, 50 μL of Buffer SDR2 (instead of 300 μL) was added during the contaminant precipitation step as recommended for low biomass soil. These three modifications, based on optional recommendations from the manufacturer, optimized RNA yield and quality for downstream applications.

The extracted RNA samples, including replicates, were sent on dry ice to Macrogen (Seoul, Republic of Korea) to prevent any potential degradation. Initial RNA quality control (QC) was conducted using the Agilent 4200 TapeStation system or 2100 Bioanalyzer with RNA ScreenTape (Agilent Technologies, Santa Clara, CA, USA) to assess the total nucleic acid quantity and the DV200 metric, which indicates the percentage of RNA fragments exceeding 200 nucleotides in length. Samples that

passed the QC criteria were used for library construction. Total RNA library preparation was performed by the sequencing company using the Illumina Stranded Total RNA Prep with Ribo-Zero Plus Microbiome kit (Illumina, San Diego, CA, USA). The constructed libraries were subsequently screened using the Agilent 2100 Bioanalyzer with HS D1000 ScreenTape (Agilent Technologies, Santa Clara, CA, USA) to measure the library concentration and size distribution. Libraries that met the QC standards were sequenced on the Illumina NovaSeq 6000 platform (Illumina, San Diego, CA, USA) at a depth of 10 Gbps, using 150 bp paired-end reads.

Method S5 Statistical analyses of metagenomic and metatranscriptomic data

Statistical analyses of metagenomic and metatranscriptomic data were also performed using R, as was done for the metabarcoding data. The relative abundance of bacterial and fungal contigs was determined by dividing read counts mapped to each contig by total read counts in each metagenomic sample. The relative abundance of the annotated genes was determined in the identical manner used in the contig-level relative abundance. The estimated relative abundance of genes was used for the ordination analyses at the functional gene level. Non-metric multidimensional scaling (NMDS) was performed with Euclidean distance estimated with the function *ordinate* in the R package phyloseq. Metagenome-based KEGG and GO term enrichment tests (differential abundance tests at the KO and GO term levels) were conducted using DESeq2 (v. 1.42.1) [7]. The analyses were conducted for three comparison pairs (Pre [n = 9] vs. Active [n = 4], Active vs. Post [n = 7], and Pre vs. Post). For these analyses, metagenomic gene abundances were agglomerated into KO or GO term levels. The resulting KO and GO term abundances were used for the enrichment tests, respectively.

Differentially expressed genes at the metagenome level were defined using metatranscriptomic read-based gene counts. Since available replicates for each soil condition were limited (two replicates per condition), the R package NOISeq (v. 2.46.0) [8], which could be applied when limited samples are available, was used. After defining metatranscriptomic DEGs, *Tm* status-specific DEGs were examined by finding DEGs commonly enriched in each condition (i.e., Active-specific DEGs: DEGs simultaneously upregulated in the Active group in both Pre-Active and Active-Post comparison pairs). Enriched GO terms were determined using the R package topGO (v. 2.54.0) [9] with *Tm* status-specific DEGs. In addition, pairwise comparison of metatranscriptomic gene expressions at the metabolic pathway level was assessed using the R package pathview (v. 1.42.0) [10].

To find microbial community-wide co-expression patterns among microbial genes, gene expression patterns were clustered using the R package Mfuzz (v. 2.62.0) [11]. We also constructed a gene co-expression network. From the TPM-normalized gene expression table, genes showing low variance across samples (threshold = 0.03069691) were filtered to exclude lowly and rarely expressed genes. A total of 558,285 genes were used as input. Using the R package WGCNA (v. 1.73) [12], gene co-expression modules (subnetworks) were predicted. Since the resulting associations predicted using WGCNA did not provide statistical significance, we estimated it by constructing a network using the SparCC algorithm implemented in FastSpar (v. 1.0.0) [13].

Method S6 Gene annotation of *T. matsutake*'s whole genome

The previously reported genome contigs of *Tm* (strain NIFoS 2001) were acquired from the NCBI Genome database (genome accession number: JALPZM000000000)[14]. The obtained contigs were soft-masked using RepeatMasker (v. 4.1.7) [15] to identify and mask repetitive elements. RNA-seq data corresponding to the same strain (SRA accession: SRR23447917) were retrieved from the NCBI Sequence Read Archive using the SRA Toolkit (v. 3.2.0)[16] and converted into paired-end FASTQ format using *fastq-dump*. Reads were aligned to the masked genome using HISAT2 (v. 2.2.1)[17], a splice-aware aligner optimized for RNA-seq data, with the parameters *--rna-strandness RF* and *--dta* to preserve strand specificity and downstream transcriptome assembly compatibility. The resulting SAM file was converted to BAM format, sorted, and quality-checked using SAMtools (v. 1.21)[18].

Gene prediction and species-specific training were conducted using BRAKER2 (v. 2.1.6)[19], which integrates GeneMark-ET[20] for RNA-seq guided gene prediction and AUGUSTUS (v. 3.4.0)[21] for evidence-supported gene model refinement. BRAKER2 was run with the sorted BAM file as RNA-seq evidence using the *--softmasking*, *--fungus*, and *--useexisting* options to generate a new AUGUSTUS species model. Final gene predictions were generated using AUGUSTUS with the trained species model and exported in GFF format with translated protein sequences. To extract gene and coding region nucleotide sequences, BEDTools (v. 2.31.1)[22] was used to convert the GFF output to BED format and retrieve sequences from the soft-masked genome. Predicted genes were further functionally annotated using emapper with EggNOG DB for consistency with the metagenomic data.

Result S1 Taxonomic composition of bacterial and fungal communities in *Tricholoma matsutake habitat* at the genus level

At the genus level, *Candidatus* Xiphinematobacter (TmD: 13.2-26.7%; TmM: 5.9-10.1%; two-sided Welch's t-test [hereafter t-test]: March, *P* = 0.3480; May, *P* = 0.3154; July, *P* = 0.0307; September, *P* = 0.0365; November, *P* = 0.4490) and *Acidothermus* (TmD: 2.9-3.6%; TmM: 0.4-1.2%; t-test: March, *P* = 0.1053; May, *P* = 0.2324; July, *P* = 0.0329; September, *P* = 0.0039; November, *P* = 0.0453), FCPS473 of Chloroflexota (TmD: 2.05-5.55%; TmM: 0.26-0.55%; t-test: March, *P* = 0.4281; May, *P* = 0.4481; July, *P* = 0.0887; September, *P* = 0.2781; November, *P* = 0.0152), and *Conexibacter* (TmD: 2.25-3.5%; TmM: 0.39-0.72%; TmM: 0.26-0.55%; t-test: March, *P* = 0.1319; May, *P* = 0.0789; July, *P* = 0.0090; September, *P* = 0.0399; November, *P* = 0.0135) were abundant in the TmD soil (Fig. S2b; Table S1). Meanwhile, AD3 of Chloroflexota (TmD: 0.92-2.95%; TmM: 4.3-11.1%; t-test: March, *P* = 0.0143; May, *P* = 0.0447; July, *P* = 0.0693; September, *P* = 0.0024; November, *P* = 0.4540), *Candidatus* Udaeobacter (TmD: 0.08-3.1%; TmM: 5.5-8.5%; t-test: March, *P* = 0.0770; May, *P* = 0.0399; July, *P* = 0.0090; September, *P* = 0.0054; November, *P* = 0.0351), and *Acidibacter* (TmD: 0.38-1.96%; TmM: 2.1-3.0%; t-test: March, *P* = 0.0351; May, *P* = 0.7604; July, *P* = 0.0004; September, *P* = 0.0128; November, *P* = 0.0128) were more abundantly detected in the TmM soil (Fig. S2b; Table S1).

In the fungal community, the distribution of genera was associated with Tm distribution patterns, although significant differences were not found due to the large abundance variations among replicates. For instance, *Sebacina* was more distributed in the TmM soil (1.1-12.2%) than in the TmD soil (0.005-4.1%) (t-test: March, *P* = 0.4645; May, *P* = 0.4645; July, *P* = 0.4645; September, *P* = 0.4645; November, *P* = 0.4645). *Russula* also showed a similar distribution pattern (TmD: 0.0-5.9%; TmM: 1.5-26.5%; t-test: March, *P* = 0.8794; May, *P* = 0.4645; July, *P* = 0.3454; September, *P* = 0.4645; November, *P* = 0.4645) (Fig. S3b; Table S1). Meanwhile, *Geminibasidium* (TmD: 6.5-15.5%; TmM: 7.8-12.8%; t-test: March, *P* = 0.5184; May, *P* = 0.5030; July, *P* = 0.4645; September, *P* = 0.5582; November, *P* = 0.4645) and *Oidiodendron* (TmD: 7.8-15.8%; TmM: 2.7-7.5%; t-test: March, *P* = 0.8547; May, *P* = 0.4645; July, *P* = 0.4645; September, *P* = 0.2039; November, *P* = 0.4645) were more abundant in the TmD soil (Fig. S3b; Table S1). In addition to the *Tm* colonization status, the bacterial and fungal communities showed a seasonal alteration. The occupancy ratio of *Candidatus* Xiphinematobacter in the TmD soil increased after July (Fig. S2b). A similar pattern was observed in Leotiomycetes (genus *Oidiodendron*) in the identical soil condition (Fig. S3b).

Result S2 Chemical environment of *T. matsutake*-colonized soils

Remarkable differences in soil chemical properties between TmD and TmM soils were

identified from TN, TP, P_2O_5, Mn, and soil water content (Fig. S4). In particular, P_2O_5 (July, 8.057 ± 5.881 mg/kg; September, 4.177 ± 3.436 mg/kg; November, 2.260 ± 0.668 mg/kg) and soil water contents (July, 8.067 ± 0.987%; September, 8.6 ± 0.9%; November, 7.567 ± 0.321%) in the TmD soil were significantly lower compared to those in the TmM soil (P_2O_5: July, 24.223 ± 4.68 mg/kg; September, 10.667 ± 1.805 mg/kg; November, 4.38 ± 0.51 mg/kg; soil water content: July, 22.867 ± 4.267%; September, 14.167 ± 0.643%; November, 10.567 ± 0.651%) (Fig. S4; Table S3). In contrast, soil Fe content was higher in the TmD soil (July, 25,958.667 ± 1,428.699 mg/kg; September, 24,874.257 ± 539.338 mg/kg; November, 26,983.837 ± 823.747 mg/kg) than in the TmM soil (July, 24,149.59 ± 310.054 mg/kg; September, 22,859.633 ± 770.04 mg/kg; November, 26,009.467 ± 361.687 mg/kg) (Fig. S4; Table S3).

Result S3 Metagenome statistics and taxonomic composition of the bacterial and fungal communities

From 20 soil samples (10 samples for 2022 and another 10 samples for 2023), we recovered a total of 33,003,858 metagenomic contigs using the mix-assembly[23] (combined assembly approach of individual assembly from each metagenomic sample and co-assembly from sample groups). The assembled contigs were further taxonomically classified using Diamond (v. 2.1.8) with the NCBI nrDB (accessed on October 25, 2023). As a result, a total of 22,381,860 bacterial and 1,088,869 fungal contigs were identified from the individually assembled contigs and co-assembled contigs. After assembling metagenomic contigs, we structurally annotated putative bacterial and fungal genes using metaprokka (v. 1.15.0) and metaeuk (v. 6.a5d39d9). We finally predicted a total of 40,440,240 bacterial and 1,224,896 fungal genes, further being clustered into 17,024,354 bacterial and 579,346 fungal representative proteins (Table S4). Among these representative genes, 13,507,113 bacterial and 490,088 fungal genes were functionally annotated using EggNog DB (v. 5.0.2).

When we examined the taxonomic composition of the bacterial and fungal communities from the individually assembled metagenomic samples, the compositional differences of microbial contigs were found. Individually assembled samples possessed 25,467 bacterial species and 2,048 fungal species (Table S4). Unidentified species belonging to Actinomycetota (5.7-25%) dominated the bacterial community in the TmD soils (Fig. S5a; Table S4). On the other hand, bacterial species affiliated to Acidobacteriota (7.3-11.7%) were more abundant in TmM soils compared to those in TmD soils (Fig. S5a; Table S4). The fungal community also showed prominent compositional differences at the metagenomic contig level. In the TmD

soils, contigs assigned to *Oidiodendron maius* (5.8-16.9%), unidentified species in Leotiomycetes (1-18.5%) and Ascomycota (7.3-19.5%) were abundant (Fig. S5b; Table S4). Meanwhile, metagenomic contigs belonging to *Metschnikowia pulcherrima* (1.6-6.3%), *Astraeus odoratus* (1.6-19.6%), *Friedmanniomyces endolithicus* (1.7-6.7%) were more distributed in the TmM soils (Fig. S5b; Table S4). The distribution of metagenomic contigs showed the successional changes in *T. matsutake* population (Fig. S4b). In 2022, *T. matsutake* dominated TmD1 (32.4%) and TmD2 (34.9%) sites, while it overwhelmed other fungal populations in the TmD1 (62.3%) and TmM1 (61.3%) sites in 2023. The population dramatically decreased in the TmD2 (1.1%) site in 2023.

Result S4 Metabarcoding data-based microbial associations

We examined whether *Tm* modulates microbial associations using network-based analysis from the metabarcoding data collected from March to November in 2022. For the TmD soil, an ASV abundance table containing 3,942 bacterial and 834 fungal ASVs was used for estimating significant associations. Meanwhile, an ASV table consisting of 4,370 bacterial and 1,620 fungal ASVs was used for the TmM soil. Significant correlations were inferred at correlation coefficient (r) values of > 0.5 or < -0.5 and a false discovery rate-adjusted $P < 0.05$. We constructed two bacterial and fungal inter-kingdom networks that consisted of 1,227 nodes (779 bacterial nodes; 448 fungal nodes) with 9,825 edges (TmD soil) and 1,770 nodes (984 bacterial nodes; 784 fungal nodes) with 10,119 edges (TmM soil), respectively (Fig. S15a; Table S15). When network complexity values were quantified, two index values (Bertz complexity and atom-bond connectivity) of the TmM soil microbial network (Bertz complexity, 36,981.82; Atom-bond connectivity, 3,541.987) were higher than those of the TmD soil microbial network (Bertz complexity, 24,694.07; Atom-bond connectivity, 2,867.785) (Table S15). These results suggest that the *Tm* dominance might lead to a decrease in the extent of microbial associations.

Tm dominance status also affected the keystone node profiles. A remarkable change was found in the roles of *Tm* in microbial associations. In the networks of the TmM soil, fungal ASVs named F2 and F3 were classified as peripherals, which have poor connectivity with others. Meanwhile, both ASVs showed a significant role as a module hub in the network of the TmD soil (Fig. S15b). These results suggest that *Tm* might be able to modulate microbial associations in the soil environments. Based on this network information, we found putative bacterial helpers or positive associates of *Tm*. In the TmD soil microbial network, compared to that of the TmM, the number of potential association partners of *Tm* increased. A total of 96 nodes (40 positive and 56

negative partners) were linked with the *Tm* nodes (Table S15). Potential positive partners in the TmD soil were assigned to *Mycobacterium*, *Burkholderia-Caballeronia-Paraburkholderia*, *Conexibacter*, *Bacillus*, *Geminibasidium*, *Humicolopsis*, *Umbelopsis*, and *Trichoderma*.

Result S5 Alteration of phage community structures by *Tricholoma matsutake* colonization

We detected 19,818 putative DNA phages from the soil metagenomes using the phage screening pipeline: filtering of metagenomic contigs with VirSorter2 and a quality check of putative phages with CheckV. Based on the instructions, we clustered the detected phages with the criteria (95% average nucleotide identity and 70% alignment rate) to obtain the representative DNA phageome, resulting in 15,355 clustered phage genomes. The clustered phage genomes were further filtered using GeNomad to classify true phage genomes. A total of 3,246 representative phages were finally recovered (Table S17). The recovered phages included a mix of complete (or near-complete) genomes and genome fragments (Fig. S16; Table S17). A total of 85 complete or high-quality (> 90% completeness) and 1,037 medium or low-quality pMAGs were confirmed to possess over ten kbp length genomes.

To figure out the effect of the colonization of *Tm* on soil phageomes, we examined the composition of DNA phages in metagenomic samples. For this purpose, we first divided the soil samples into three groups according to the *Tm* colonization history (Active, soils where *Tm* was actively colonized; Pre, soils where Tm was not colonized yet; Post, soils where *Tm* was colonized past). The composition of DNA phages in soil samples showed the dramatic domination of Caudoviricetes irrespective of the colonization status of *Tm* (Fig. S17a). Although the class-level composition of the soil phageome did not show significant differences across samples according to the *Tm* colonization, an ordination analysis showed the clustering of soil phageomes by the same factor, suggesting the compositional alteration in the soil phageomes (Fig. S17b). PERMANOVA further corroborated the significant effect of *Tm* colonization on the composition of soil phageomes ($R^2 = 0.67441$, $P = 0.001$). Based on these results, we next aimed to find DNA phages involved in the observed compositional differences among the *Tm* colonization status. For this purpose, we compared abundance patterns of the 3,246 soil phages across the soil samples using a distribution heat map. Based on the metagenomic read-based phage abundance, soil samples were clearly clustered together depending on the Tm colonization status except for one sample (Y23TmM1) (Fig. S17c). The Y23TmM1 sample was designated

as the active site where *Tm* was actively colonized based on metagenomic and metatranscriptomic abundances of *Tm*. It was closely clustered with pre-colonization samples (Fig. S17c).

Fig. S1

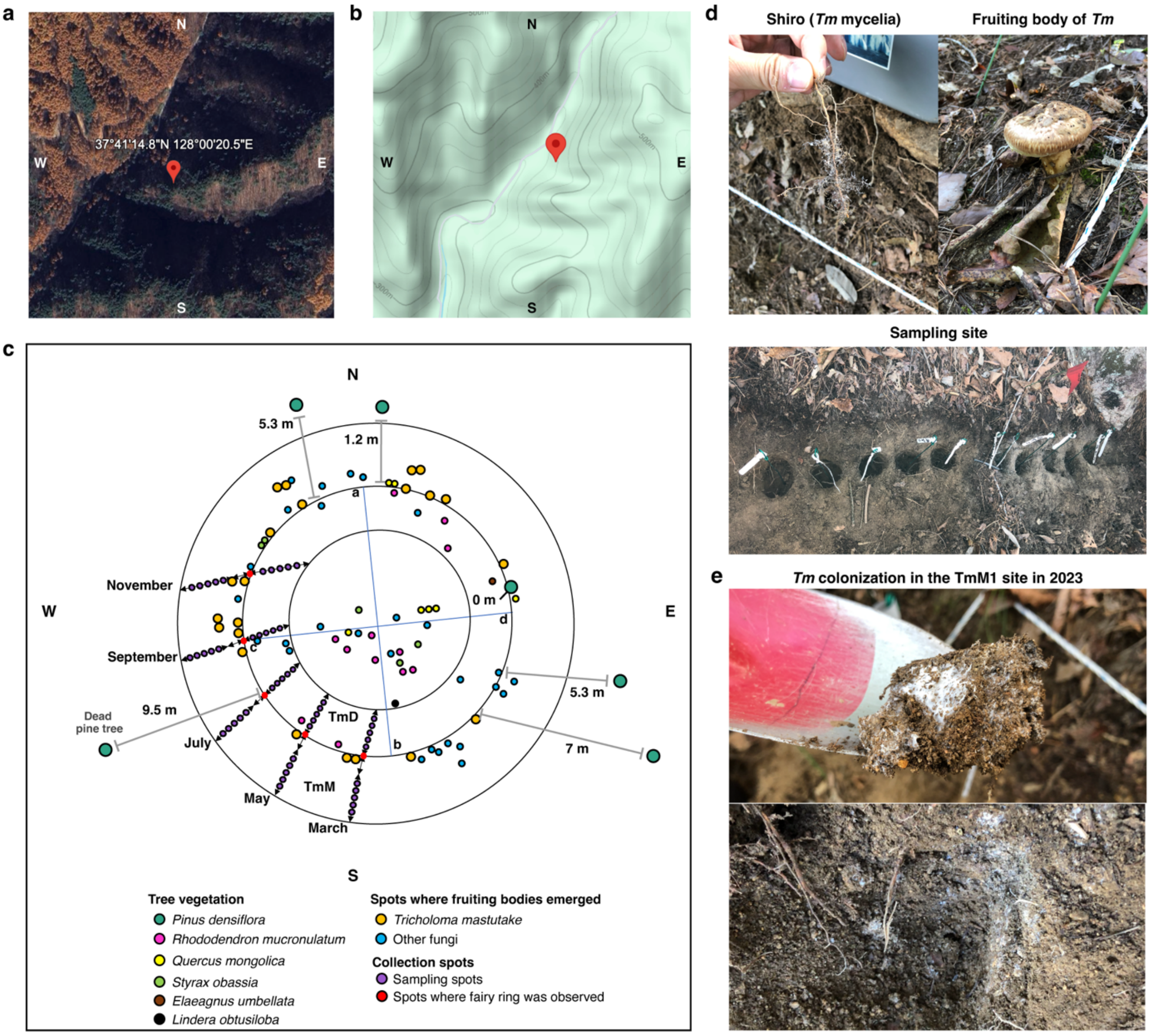

Fig. S1. Soil collection spots and vegetation distribution in the sampling area. a, A satellite view of the sampling area. **b**, A terrain map showing the elevation of the sampling area. The red pins in panels **a** and **b** indicate the actual sampling area. **c**, Vegetation diagram in the sampling area. **d-e**, Field observation of Tricholoma matsutake in dominant and minor soil zones. **d**, *T. matsutake* mycelial aggregates attached to fine roots of pine, observed in the dominant zone (TmD1) (top left panel of Fig. S1d); Fruiting body arising in September 2022 near the marked fairy ring area (outlined with white rope) (top right panel of Fig. S1d); Layout of the 10 soil sampling points, with minor (TmM) zones on the left and dominant (TmD) zones on the right relative to the fairy ring edge (bottom panel of Fig. S1d). **e**, Spider web–like mycelial aggregates in soil from the first minor sampling point (TmM1) in September 2023 (top panel of Fig. S1e); White powdery area on the right side of the TmM1 soil (facing the 2022 fairy ring edge), indicating *T. matsutake* mycelial growth (bottom panel of Fig. S1e). The diagram shows the fairy ring of *Tricholoma matsutake* originating from a single fungus, represented by a circle labeled a, b, c, and d. The purple dots represent

the sampling spots, and the red dots represent the center of the fungus. TmD and TmM samples were assigned numbers 1 to 5 from the nearest to the center of the fungus. We sampled every two months at intervals of 1 m across the circumference of the site and collected TmM soil samples at 20 cm intervals from the center of the fungus. The sampling spots, with five replicates, were taken at 10 cm intervals each. The range of sampling is 75 cm, which is the sum of five repetitions of seven cm across the soil and four repetitions of 10 cm between sampling spots. Each green circle represents a pine tree (*Pinus densiflora*). Each orange circle represents a fruiting body of *Tm*. Each blue circle represents the fruiting bodies of other fungi that appeared inside and around the fairy ring. The species include *Marasmius maximus* Hongo, *Marasmius siccus*, *Mycena sanguinolenta* (Alb. & Schwein.) P. Kumm., *Clitocybe cyanophaea* (Fr.) Gillet, *Gymnopus peronatus* (Bolton) Antonin, Halling & Noordel., *Inocybe asterospora* Quel., *Marasmius oreades* (Bolton) Fr., *Marasmius leveilleanus* (Berk.) Sacc., *Russula mariae* Peck, *Amanita pantherina*, *Marasmius confertus* var. tenuicystidiatus Antonin, *Russula lilacea* Quel., *Strobilomyces confusus* Sing., *Russula emetica* (Schaeff.) Pers., *Marasmius graminum* (Lib.) Berk., *Psathyrella candolleana*, *Podoscypha nitidula* (Berk.) Pat., *Collybia dryophila* (Bull.:Fr) Kummer, *Hydnellum concrescens* (Pers.) Banker, and *Helvella crispa* (Scop.) Fr. The magenta (*Rhododendron mucronulatum*), light yellow (*Quercus mongolica*), light green (*Styrax obassia*), brown (*Elaeagnus umbellata*), and black (*Lindera obtusiloba*) circles represent tree species. TmD, *T. matsutake*-dominant soils; TmM, *T. matsutake*-minor soils.

Fig. S2

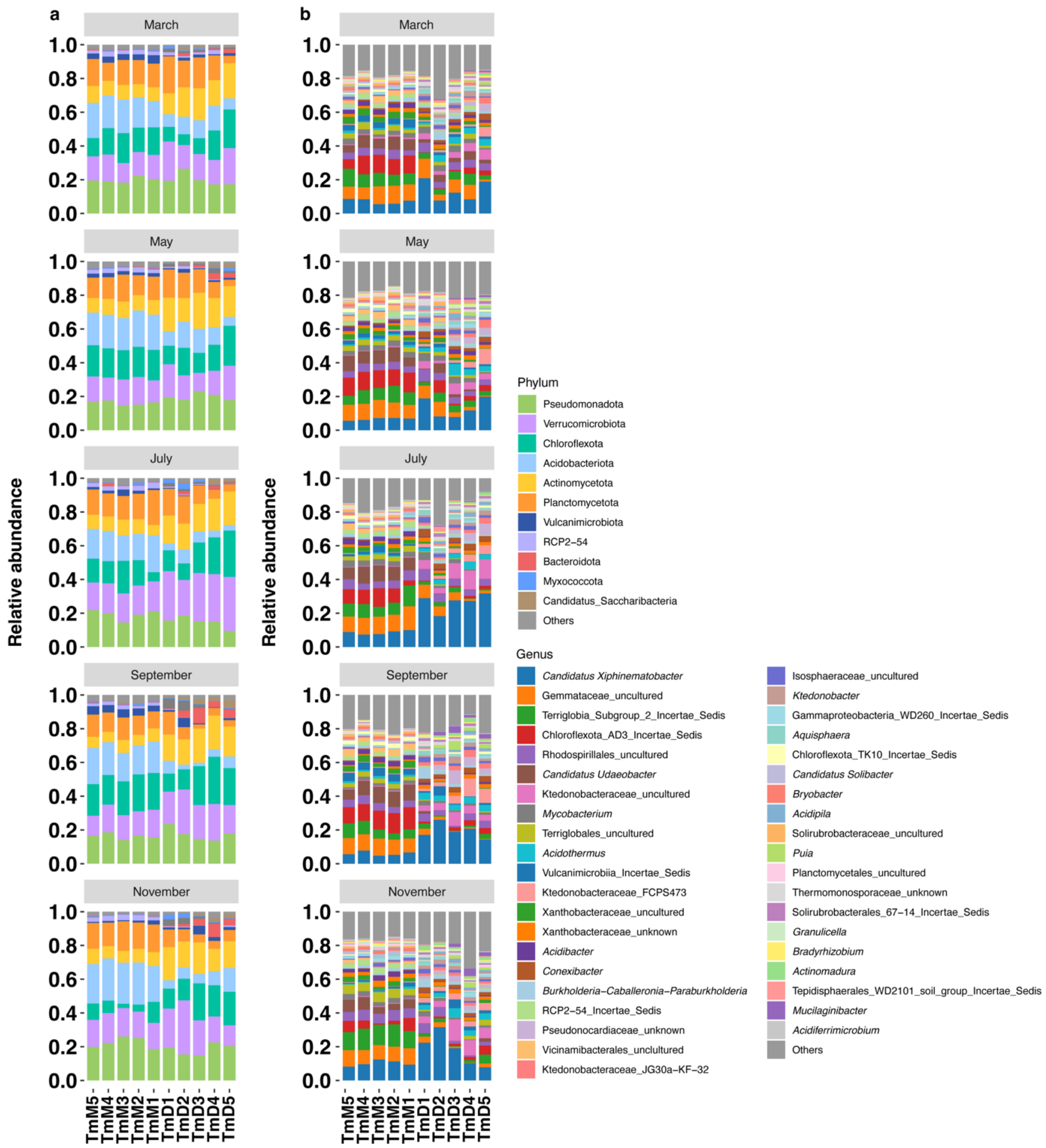

Fig. S2. The composition of bacterial communities in each sampling site from March to November. a, The composition at the phylum level. **b**, The composition at the genus level. The exact relative abundance values are available in Table S1.

Fig. S3

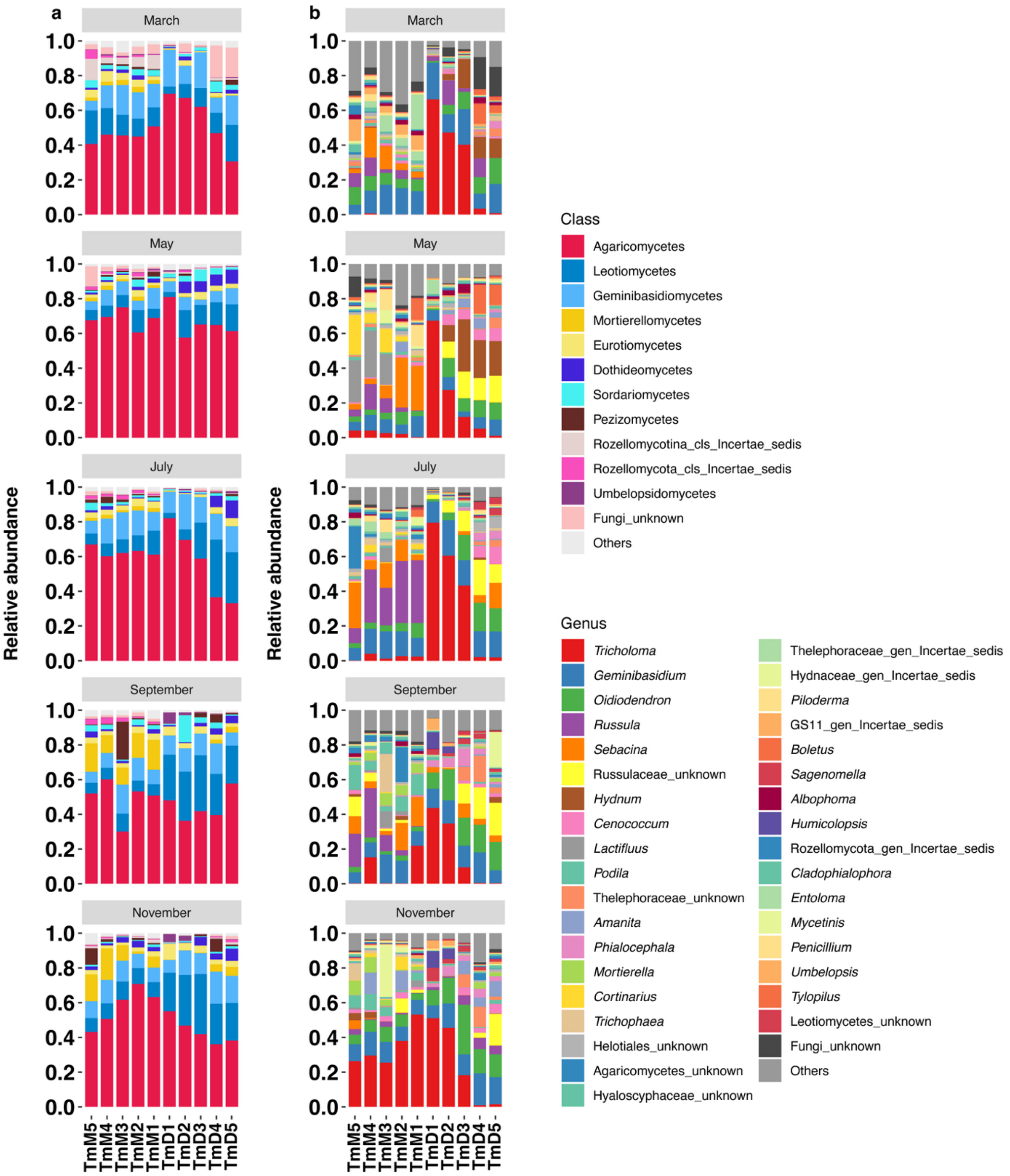

Fig. S3. The composition of fungal communities in each sampling site from March to November. a, The composition at the phylum level. **b**, The composition at the genus level. The exact relative abundance values are available in Table S1.

Fig. S4

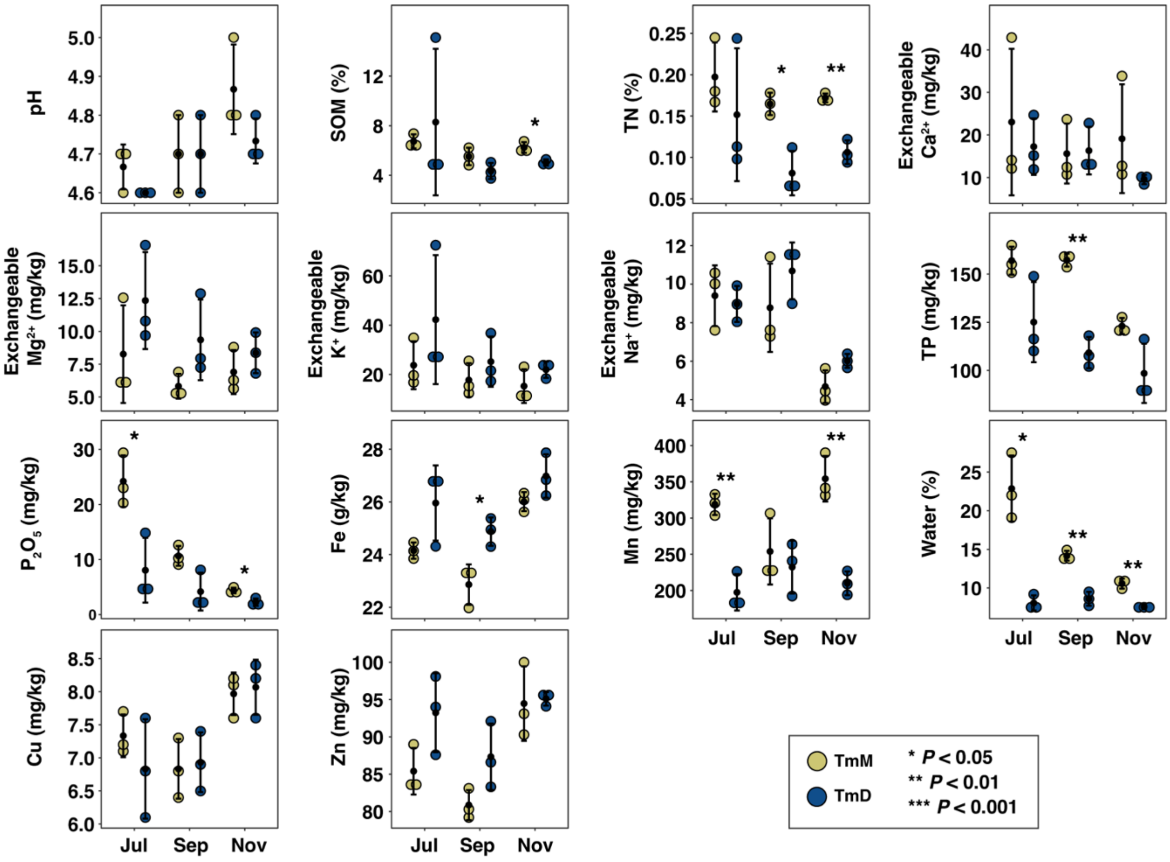

Fig. S4. Soil chemical properties of *Tricholoma matsutake*-dominant and minor soils. Dots correspond to exact values of soil chemical properties each month. According to the normality test results, the Student's t-test was performed for each chemical property. Asterisks indicate significant differences in soil chemical properties between TmM (*T. matsutake*-minor soil) and TmD (*T. matsutake*-dominant soil). The data on the statistical analyses are available in Table S3.

Fig. S5

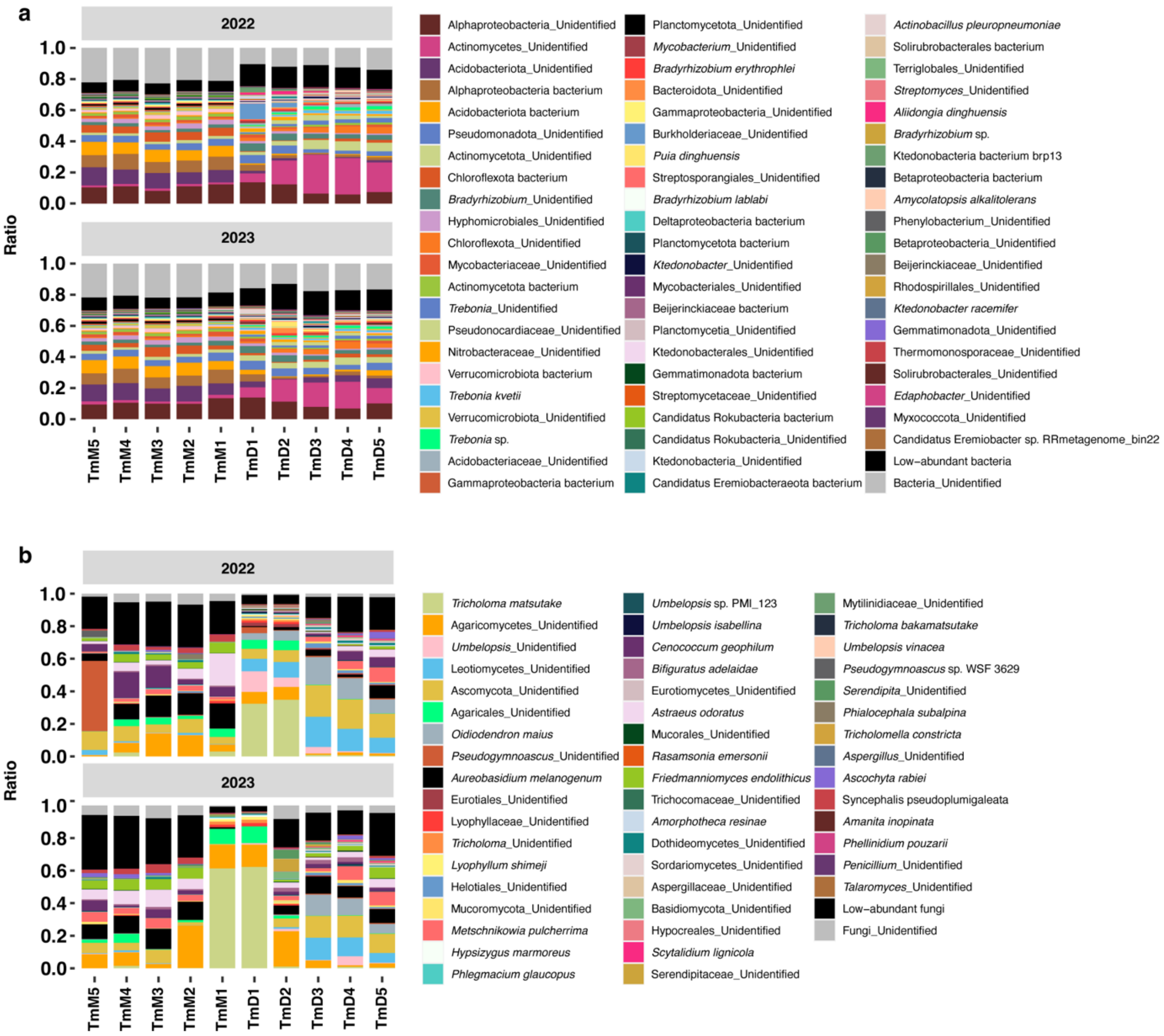

Fig. S5. Taxonomic composition of metagenomic contigs retrieved from September soil samples. a, The composition of bacterial contigs. **b**, The composition of fungal contigs. Metagenomic contigs were taxonomically classified using Diamond (v.) with the NCBI non-redundant DB (accessed on October 25, 2023). Bacterial and fungal contigs were obtained based on the taxonomy classification results. The ratio of the contigs in each domain was calculated by dividing the read counts mapped to each contig by the total mapped read counts. TmD, *Tricholoma matsutake*-dominant soils; TmM, *T. matsutake*-minor soils. The exact values for this figure are available in Table S4.

Fig. S6

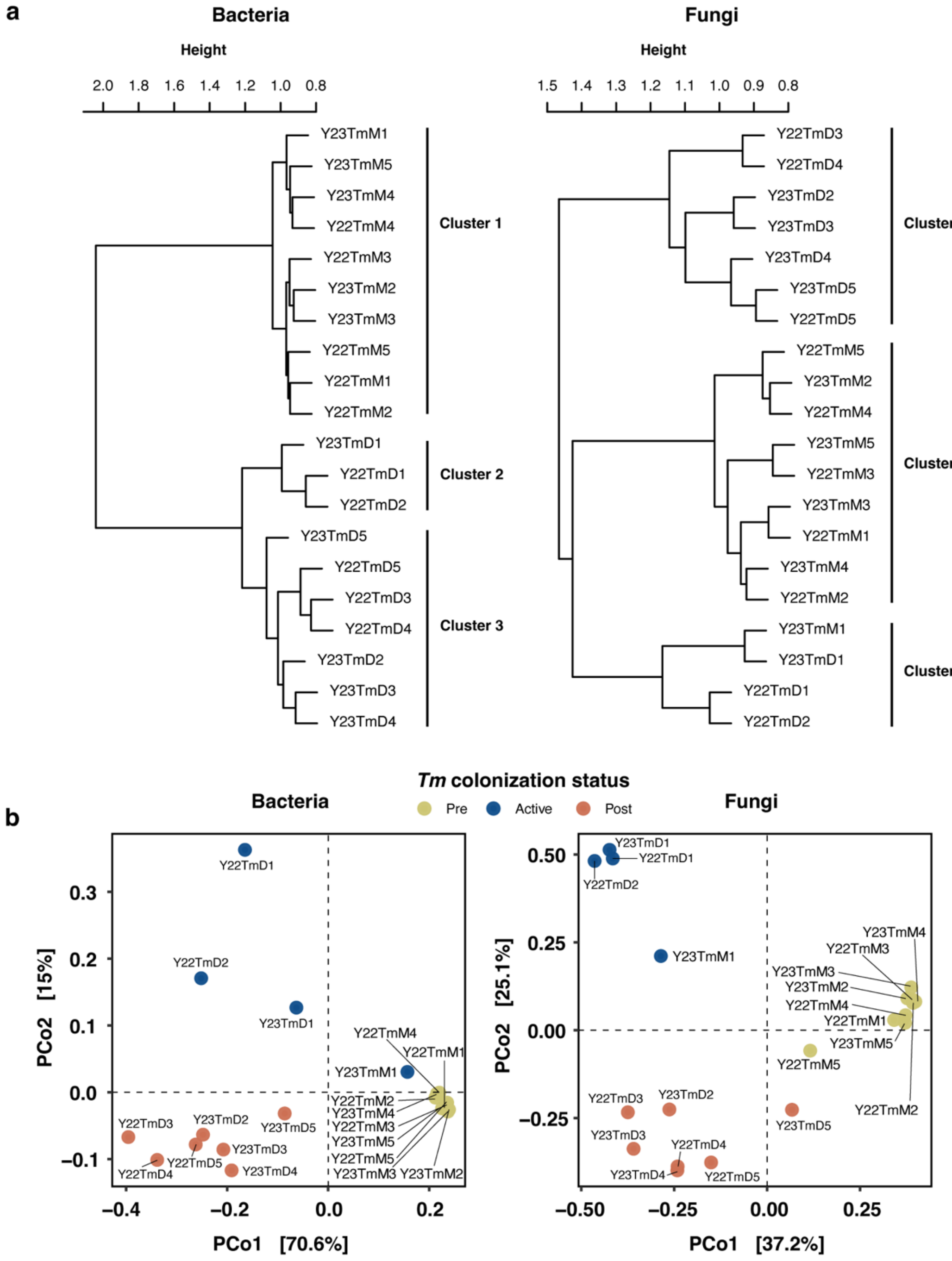

Fig. S6. The clustering analyses of bacterial and fungal metagenomic samples. a, Hierarchical clustering of the bacterial and fungal metagenomic samples. Hierarchical clustering at the metagenomic sample level was performed using 1-Pearson correlation with the Ward linkage method. Sample clusters were found based on the K-means clustering. **b**, Unconstrained principal coordinate analysis of the bacterial and fungal metagenomic samples. Cumulative sum scaling (CSS)-normalized abundance data were used to estimate Bray-Curtis distance values. Dots

indicate each metagenomic sample, and they are colored based on the *Tm* colonization status.

Fig. S7

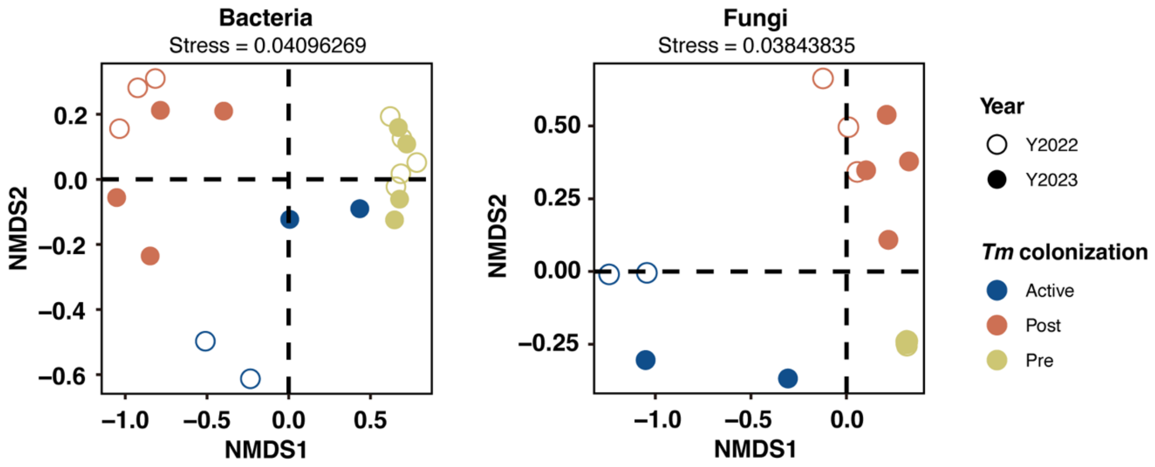

Fig. S7. Ordination analysis of the functional diversity of soil bacterial and fungal communities. The gene abundances are determined by the read counts mapped to the annotated genes. For the ordination analysis, the relative abundance of genes was used. Dots indicate each metagenomic sample, and they are colored by the Tm colonization status. Hallow dots demonstrate the samples collected in 2022, whereas filled dots show those collected in 2023.

Fig. S8

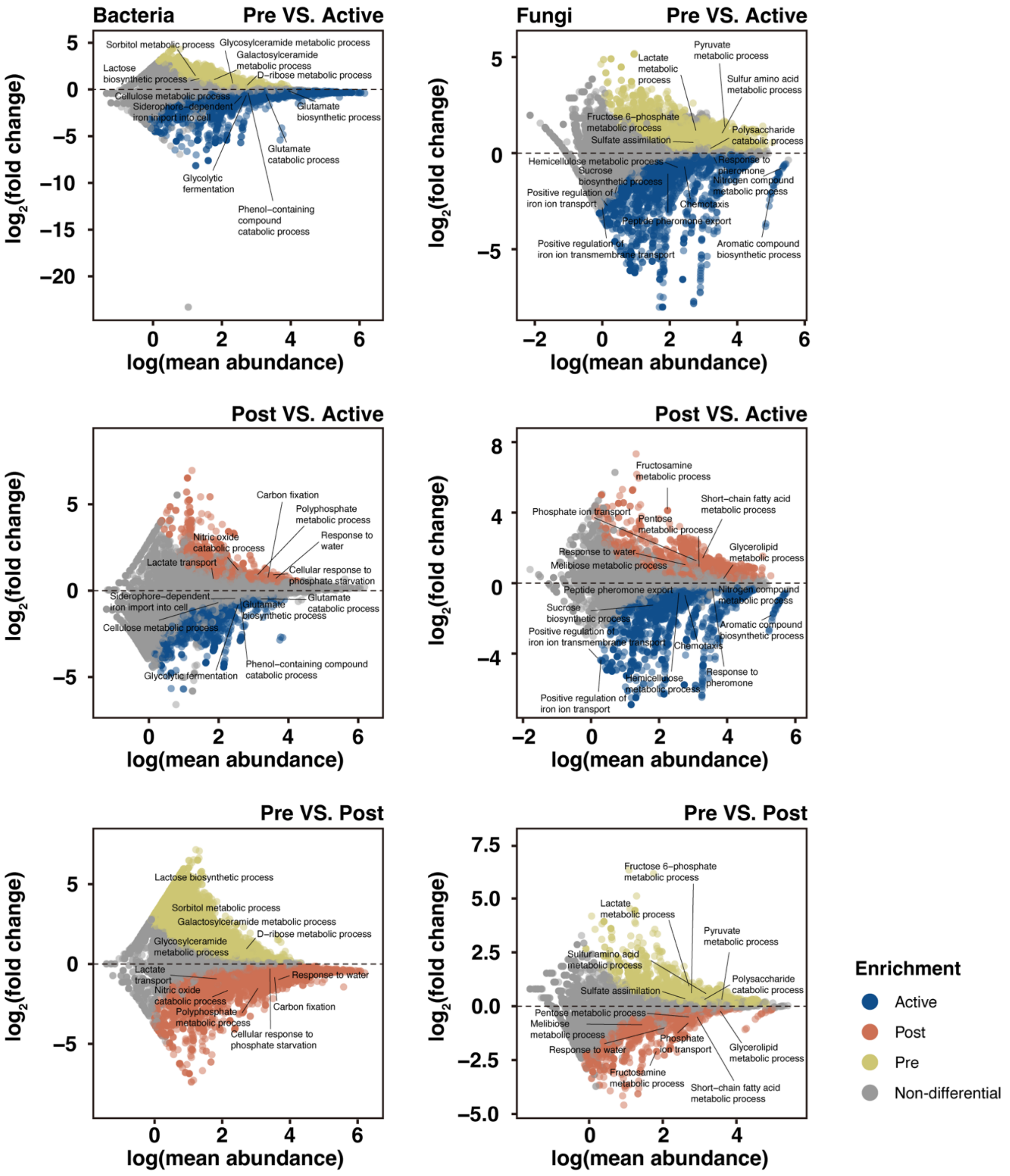

Fig. S8. Differential abundance assessment at the gene ontology (GO) term level. Differential abundance assessment was performed based on the DESeq2-normalized abundance of GO terms. The abundance of GO terms was estimated by summing the read counts of the annotated genes at the GO term level. Significantly abundant GO terms were defined as those showing log2 fold change > 0 or < 0 and FDR-corrected P < 0.05 in pairwise comparison pairs (Pre vs. Active; Active vs. Post; Pre vs. Post).

Fig. S9

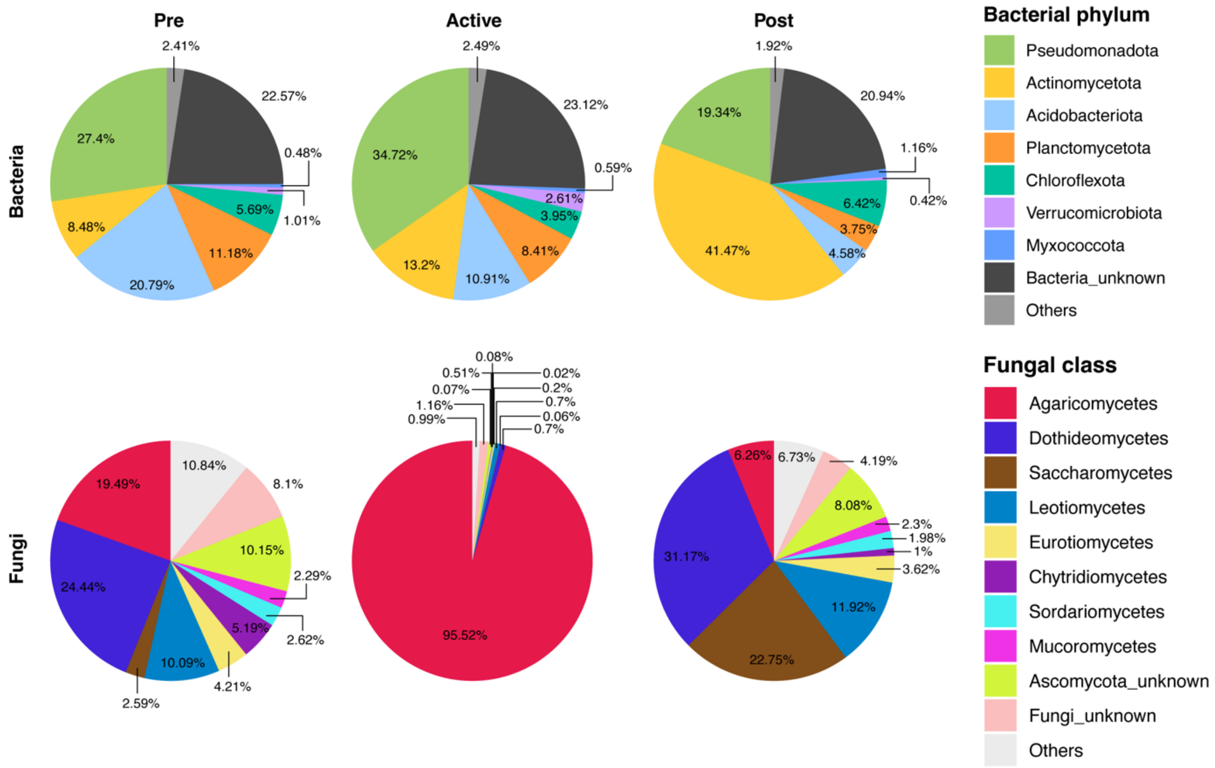

Fig. S9. Taxonomic affiliation of specifically upregulated genes in each *Tricholoma matsutake* colonization status at the phylum or class level. The pie charts show the taxonomic affiliation of specifically upregulated genes in each *T. matsutake* condition. Others consist of low-abundant phyla or classes whose ratio is lower than 0.005. Pre, genes upregulated explicitly in the Pre group; Active, genes specifically upregulated in the Active group; Post, genes upregulated explicitly in the Post group.

Fig. S10

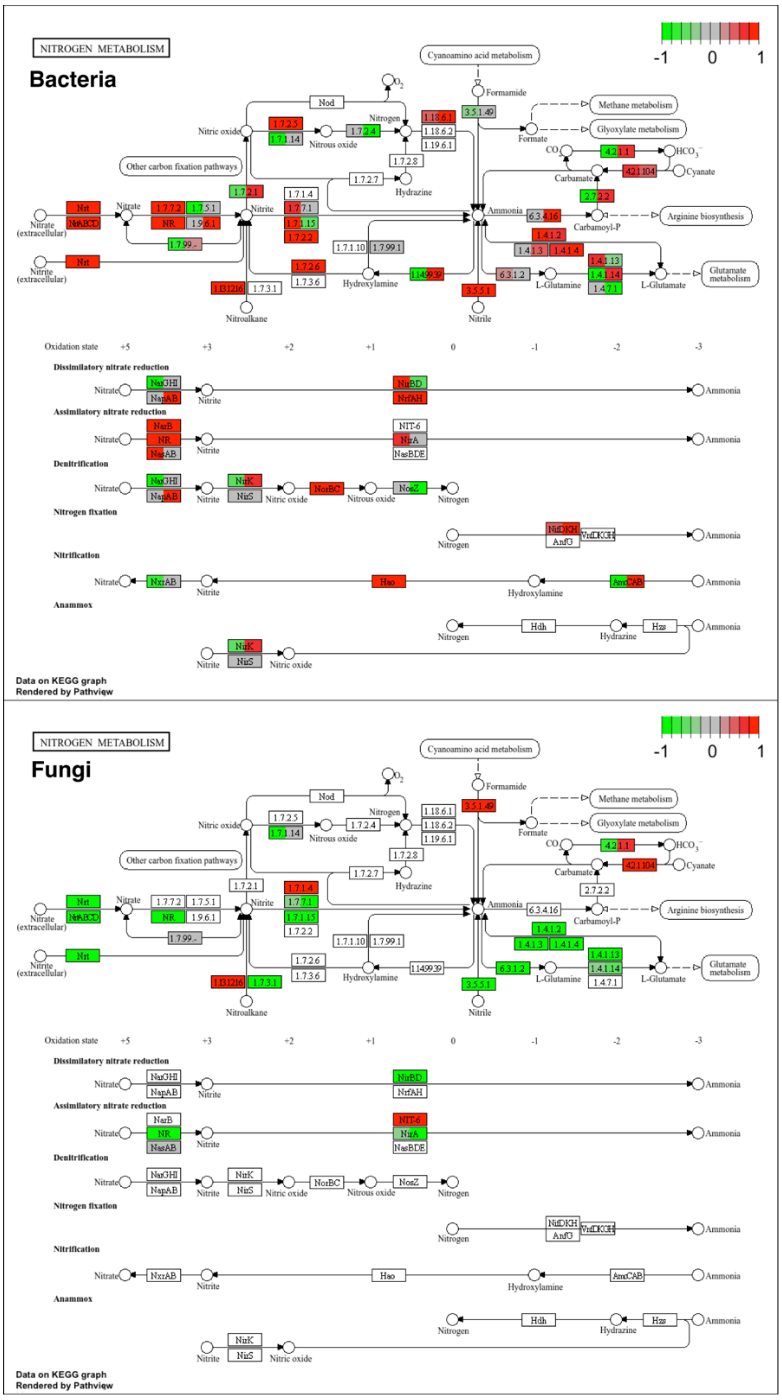

Fig. S10. Differential expression of KEGG orthologs (KOs) involved in the nitrogen metabolism in the bacterial and fungal communities. The expression pattern of the bacterial community is depicted on the top panel, whereas that of the fungal community is on the bottom panel. The KEGG pathway was visualized using the R package Pathview. The color gradient indicates the $\log_2$ fold change values estimated from the pairwise comparison pairs (Active vs. Pre and Active vs. Post). Red colors indicate that the expression of KOs is upregulated in the Active group, whereas green colors represent that that of KOs is downregulated.

Fig. S11

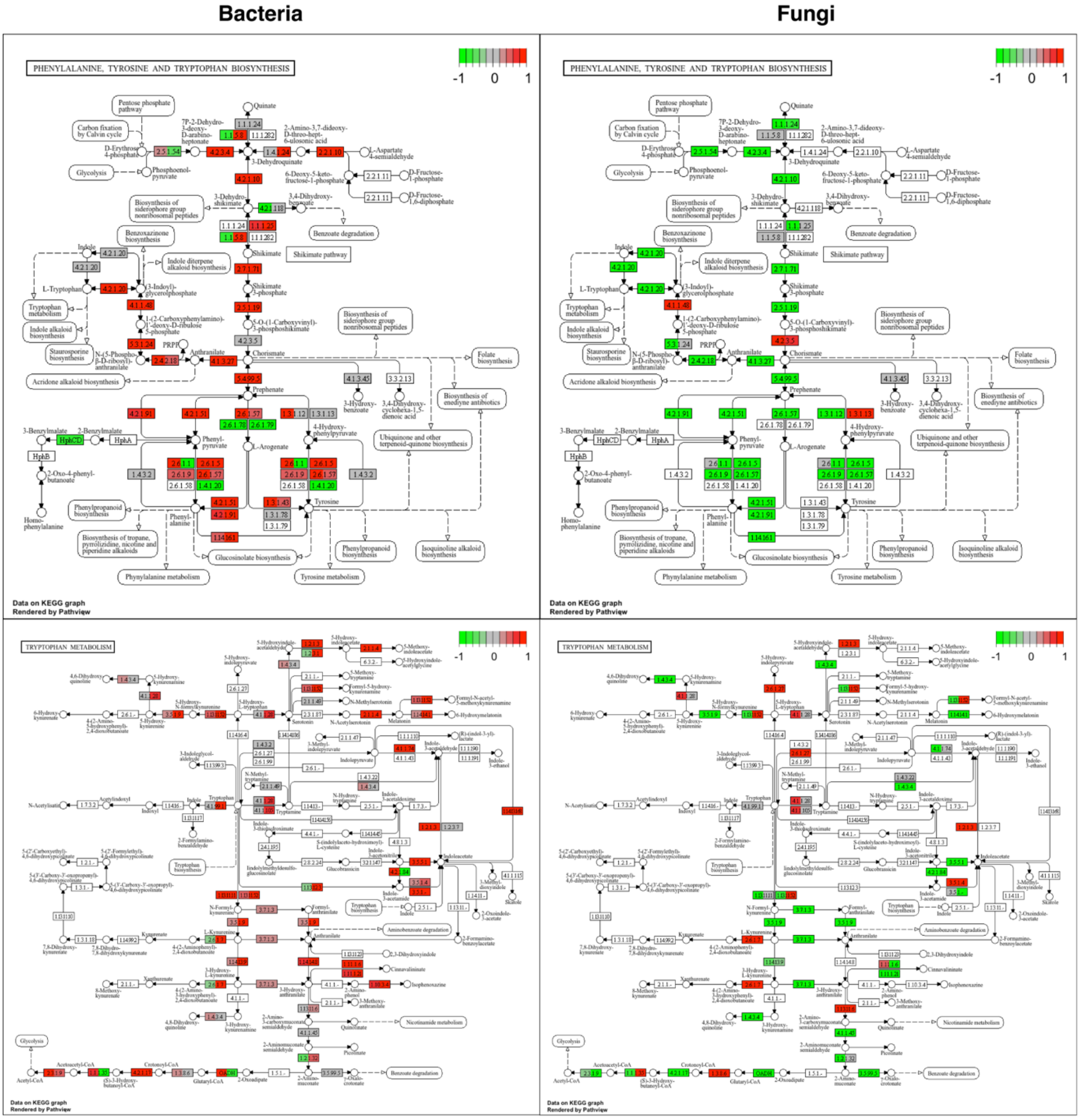

Fig. S11. Differential expression of enzymes involved in the tryptophan biosynthesis and metabolism in the bacterial and fungal communities. The KEGG pathways are visualized using the R package Pathview. The color gradient indicates the $\log_2$ fold change values estimated from the pairwise comparison pairs (Active vs. Pre and Active vs. Post). Red colors indicate that the expression of KOs is upregulated in the Active group, whereas green colors represent that that of KOs is downregulated.

Fig. S12

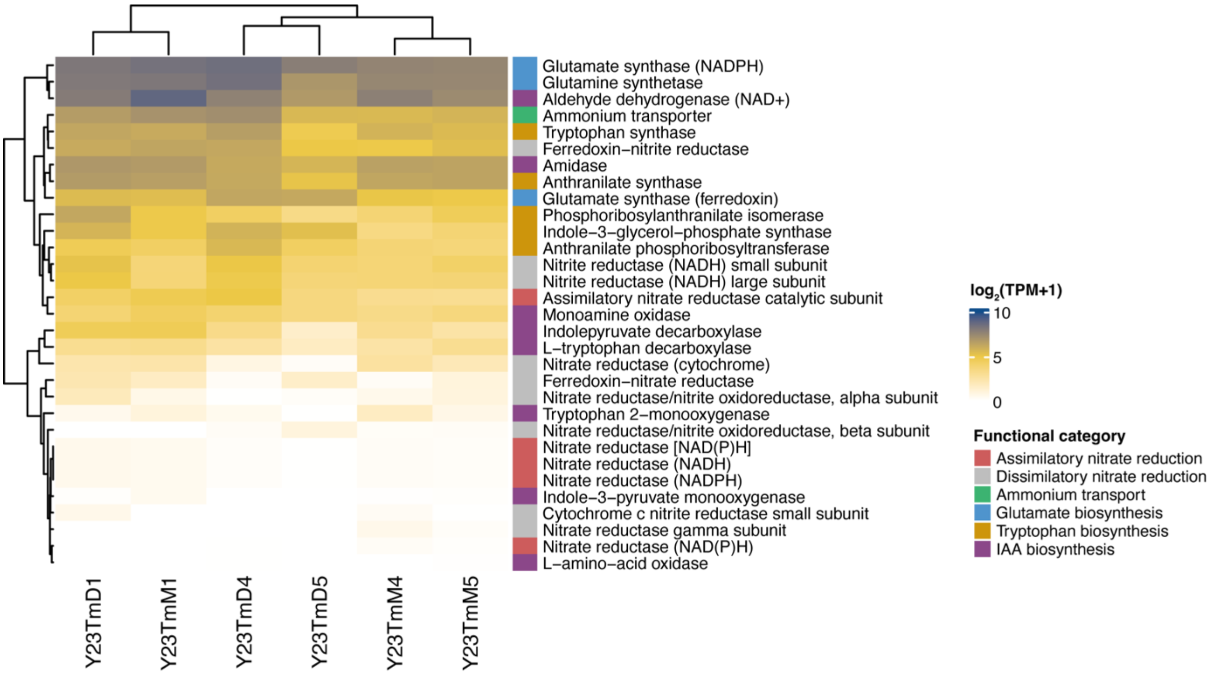

Fig. S12. Expression of bacterial genes involved in nitrogen metabolisms in the context of IAA biosynthesis. Rows and columns correspond to bacterial genes and metatranscriptomic samples, respectively. The colors of the tiles indicate the expression level of each gene, which was log2-transformed TPM+1 values. Hierarchical clustering of rows and columns was conducted with the maximum algorithm. The squares next to the heat map represent functions or metabolic pathways in which each gene participates.

Fig. S13

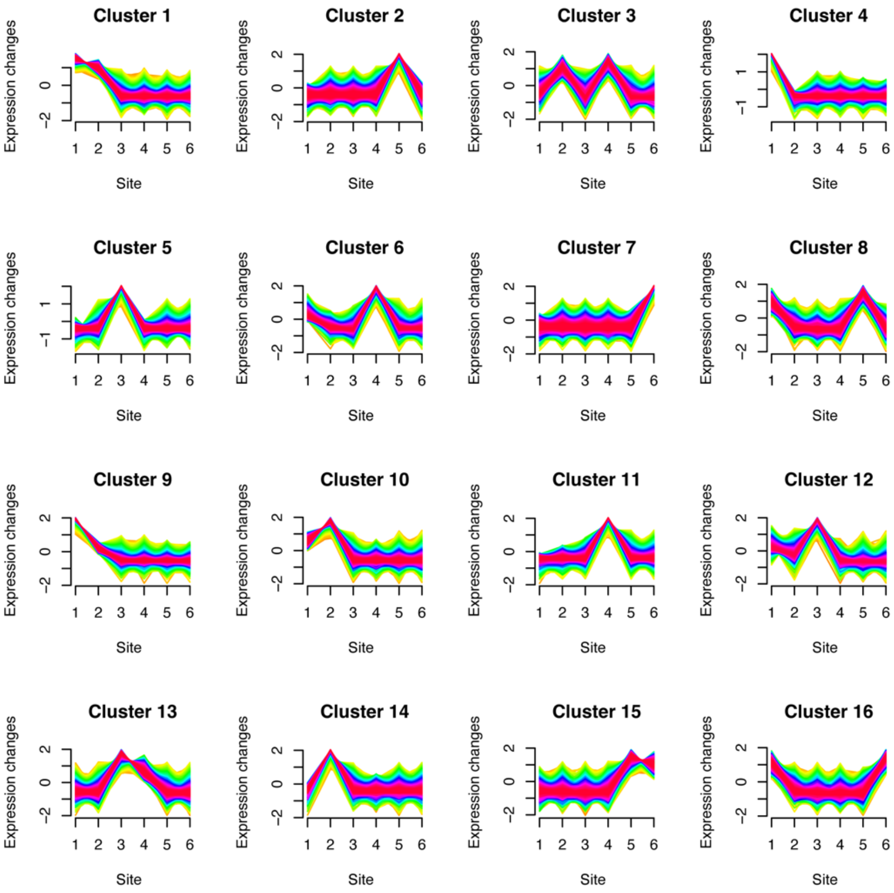

Fig. S13. Clustering of bacterial and fungal gene expression patterns. TPM values of bacterial and fungal genes were used for the expression pattern clustering. The expression patterns were clustered using the R package Mfuzz (v. 2.62.0). The numbers on the x-axis indicate the soil sites (1, TmM5; 2, TmM4; 3, TmM1; 4, TmD1; 5, TmD4; 6, TmD5). The color code reflects Membership values calculated by Mfuzz, where red corresponds to high values and green to low values of Membership score.

Fig. S14

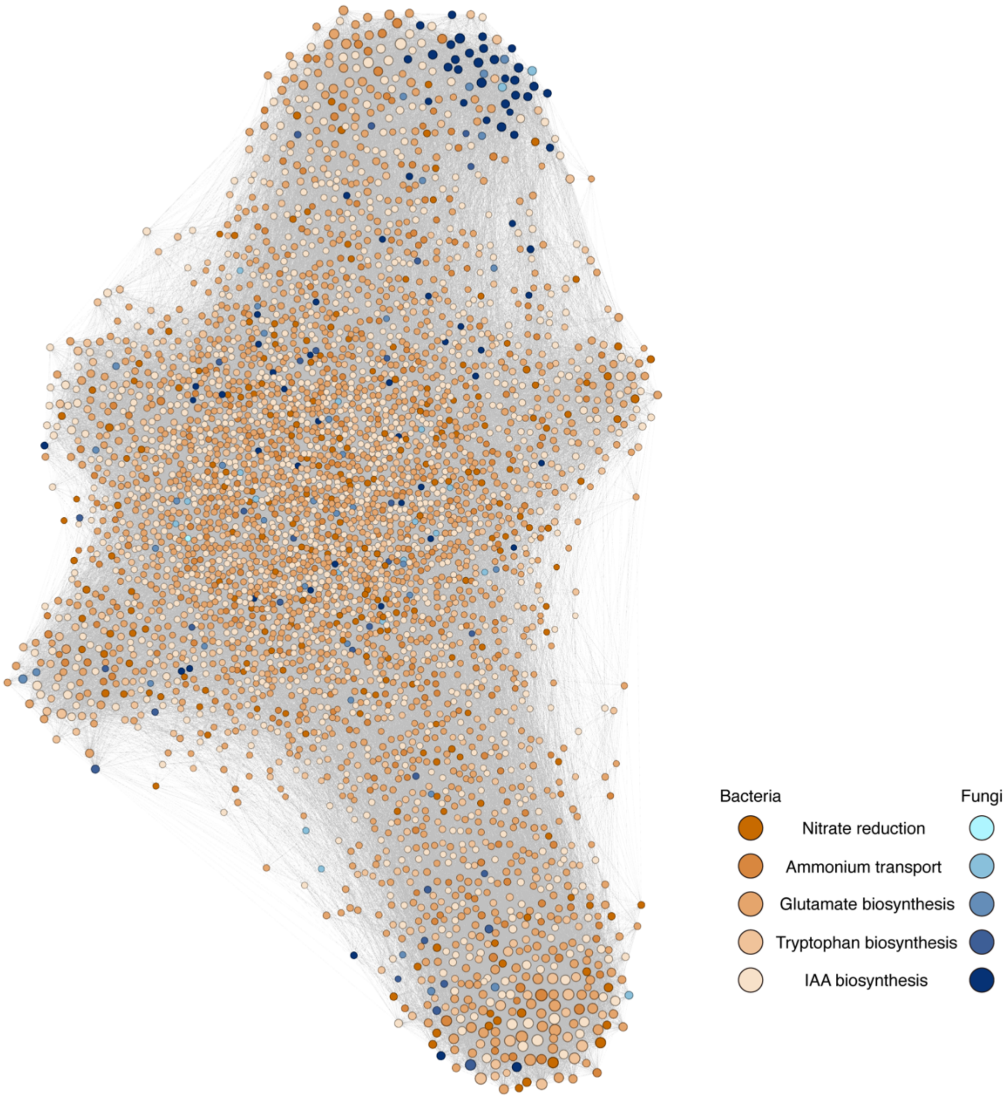

Fig. S14. Co-expression network of bacterial and fungal genes participating in the indole-3-acetic acid (IAA) biosynthesis. For constructing the gene co-expression network, a TPM-normalized gene expression table was used as an input. The pairwise correlation among genes was estimated with the SparCC algorithm implemented in FastSpar (v. 1.0.0). Significant co-expression patterns were filtered based on the criterion: correlation coefficient value > 0.2 and pseudo-P < 0.05. Each node corresponds to each gene involved in the IAA biosynthesis. Node colors indicate the functional categories of the genes and the microbial domain possessing each gene.

Fig. S15

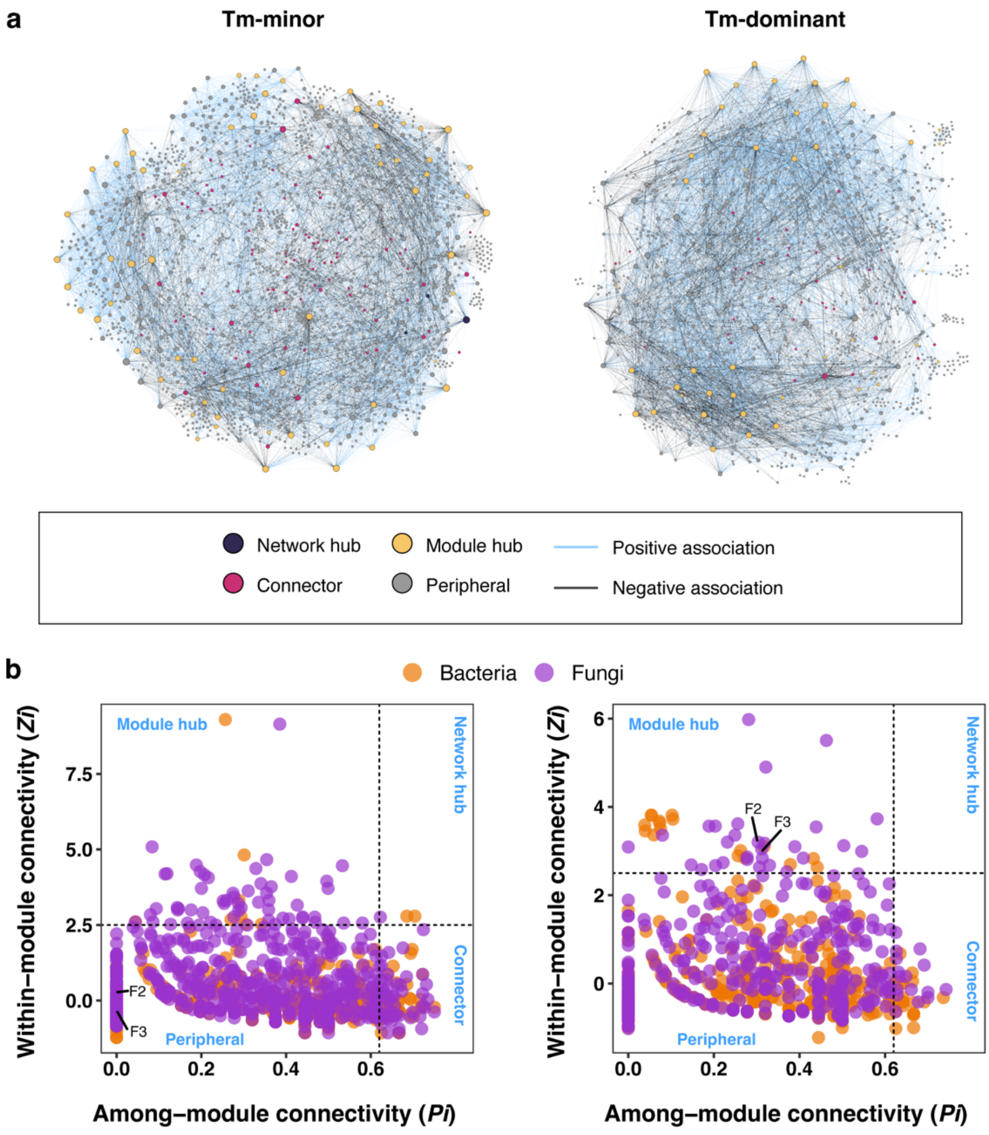

Fig. S15. Metabarcoding-based microbial associations in the *Tricholoma matsutake* habitat. Bacterial-fungal associations in the Tm-minor (right panel) and dominant (left panel) soils. The colors of nodes represent the roles of each node in the associations (dark blue, network hub; yellow, module hub; violet-red, connector; gray, peripheral). The line colors indicate positive (light blue) and negative (black) associations. b, Keystone nodes of Tm-minor (left panel) and dominant (right panel) soil microbial communities. Each circle corresponds to each node of the associations. Circle colors indicate microbial kingdoms where each node belongs (orange, bacteria; purple, fungi). Keystone nodes are predicted using within (Zi)- and among-module (Pi) connectivity (network hub, Zi ≥ 2.5 and Pi ≥ 0.62; module hub, Zi ≥ 2.5

and $Pi < 0.62$; connector, $Zi < 2.5$ and $Pi \geq 0.62$; peripherals, $Zi < 2.5$ and $Pi < 0.62$). Nodes identified as *Tricholoma matsutake* (F2 and F3) are shown in each panel.

Fig. S16

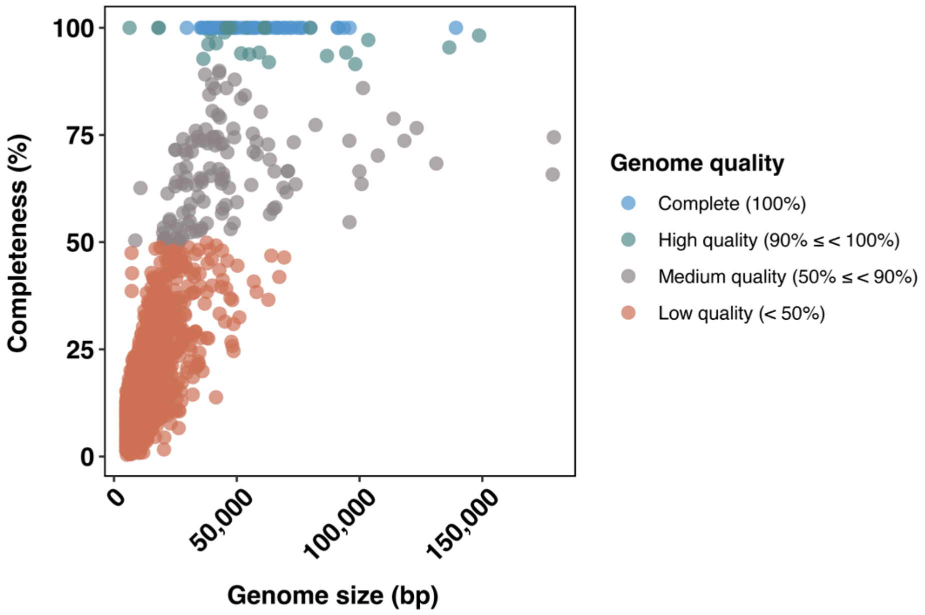

Fig. S16. The genome size and completeness of representative DNA phages. The completeness of representative DNA phages was estimated using checkV (v. 1.0.1). Dots are colored by the quality of the corresponding phages. A total of 479 phages whose quality was not determined were not presented in the figure. The exact values for genome sizes and completeness are available in Table S15.

Fig. S17

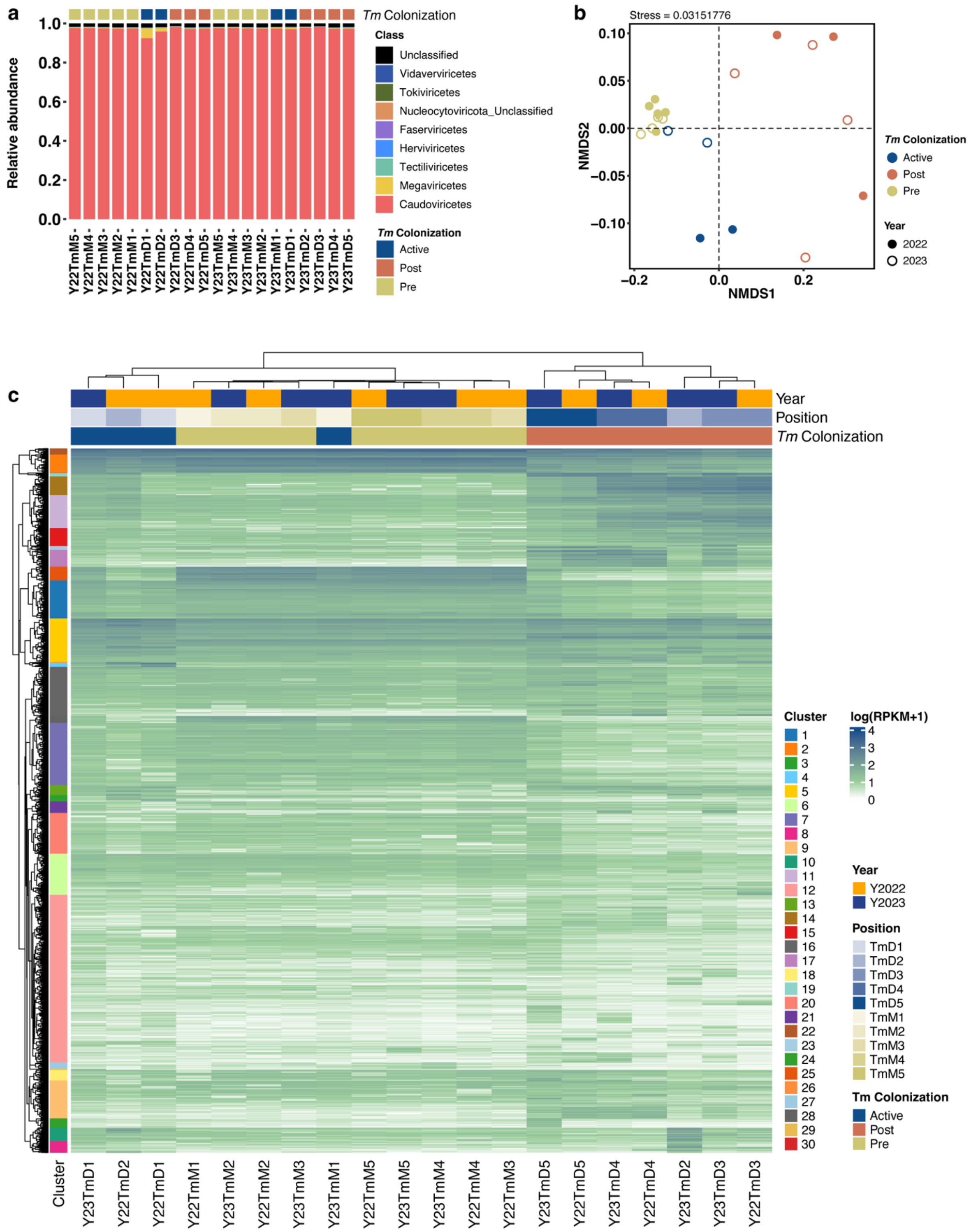

Fig. S17. Composition and distribution of DNA phages in *T. matsutake*-dominant and minor soils. a, Taxonomic composition of DNA phages at the class level. Bars are colored by viral classes. The squares above the bars indicate the colonization status of

T. matsutake (Active, blue; Pre, yellow; Post, red). Each tick of the *x*-axis corresponds to each sample. Relative abundance of each viral class was estimated by dividing metagenomic read counts mapped to each pMAG by the total mapped metagenomic read counts in discrete samples. **b**, Ordination plot of DNA phageomes. The ordination analysis was conducted using NMDS with Bray-Curtis distance. The distance among samples was calculated based on the reads per kilobase of transcript per million mapped reads (RPKM) values of phageomes. Each dot corresponds to each sample and is colored by the colonization status of *T. matsutake* (Active, blue; Pre, yellow; Post, red). The shapes of dots indicate the years when samples were obtained (2022, filled; 2023, hollow). **c**, Distribution heat map of DNA phageomes in the examined samples. pMAGs in the rows and samples in the columns were clustered using the maximum and Pearson correlation algorithm, respectively. The colors of cells indicate the log-transformed RPKM+1 values of each DNA phage in samples. Column annotations above the heat map include collecting years (2022, orange; 2023, navy), soil position (*Tm*-minor sites, yellow; *Tm*-dominant sites, blue), and colonization status of *T. matsutake* in each sample (Active, blue; Pre, yellow; Post, red). Rows were annotated with the 20 clusters of pMAGs showing similar distribution patterns across the examined samples. Y22, samples collected in September 2022; Y23, samples collected in September 2023; TmD, *T. matsutake*-dominant soil; TmM, *T. matsutake*-minor soil. Numbers (1, 2, 3, 4, and 5) indicate positions where each sample was obtained.

Fig. S18

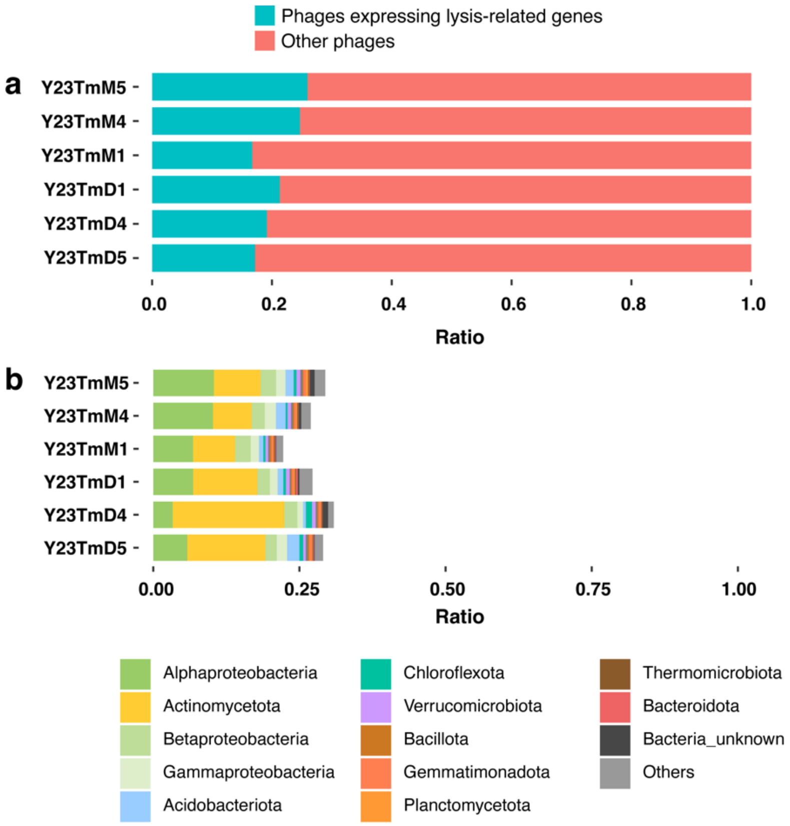

Fig. S18. Ratio of active lytic phages and the relative abundance of their bacterial hosts at the genus level. a, Ratio of phages expressing lysis-related genes and other phages. The ratio values in each sample were calculated by dividing the numbers of each phage group in each sample by the total number of phages (n = 3,246). **b**, Relative abundance of the phages consistently expressing lysis-related genes across all samples. The relative abundance values were calculated by dividing reads mapped to the identified active lytic phages by the total reads of each sample.

References

1. Walkley A, Black IA. An examination of the Degtjareff method for determining soil organic matter, and a proposed modification of the chromic acid titration method. Soil Sci. 1934;37:29–38; doi: 10.1097/00010694-193401000-00003.
2. Bremner J, Jenkinson D. Determination of organic carbon in soil .I. Oxidation by dichromate of organic matter in soil and plant materials. J Soil Sci. 1960;11:394–402; doi: 10.1111/j.1365-2389.1960.tb01093.x.
3. Bray RH, Kurtz LT. Determination of total, organic, and available forms of phosphorus in soils. Soil Sci. 1945;59:39–45; doi: 10.1097/00010694-194501000-00006.
4. Caporaso JG, Lauber CL, Walters WA, Berg-Lyons D, Lozupone CA, Turnbaugh PJ, et al. Global patterns of 16S rRNA diversity at a depth of millions of sequences per sample. Proc Natl Acad Sci U S A. 2011;108:4516–22; doi: doi:10.1073/pnas.1000080107.
5. Lundberg DS, Yourstone S, Mieczkowski P, Jones CD, Dangl JL. Practical innovations for high-throughput amplicon sequencing. Nat Methods. 2013;10:999–1002; doi: 10.1038/nmeth.2634.
6. Op De Beeck M, Lievens B, Busschaert P, Declerck S, Vangronsveld J, Colpaert JV. Comparison and validation of some ITS primer pairs useful for fungal metabarcoding studies. PLoS One. 2014;9:e97629; doi: 10.1371/journal.pone.0097629.
7. Love MI, Huber W, Anders S. Moderated estimation of fold change and dispersion for RNA-seq data with DESeq2. Genome Biol. 2014;15:550; doi: 10.1186/s13059-014-0550-8.
8. Tarazona S, García-Alcalde F, Dopazo J, Ferrer A, Conesa A. Differential expression in RNA-seq: a matter of depth. Genome Res. 2011;21:2213–23; doi: 10.1101/gr.124321.111.
9. Alexa A, Rahnenführer J. Gene set enrichment analysis with topGO. Bioconductor Improv. 2009;27:776. Accessed 16 August 2024
10. Luo W, Brouwer C. Pathview: an R/Bioconductor package for pathway-based data integration and visualization. Bioinformatics. 2013;29:1830–1; doi: 10.1093/bioinformatics/btt285.
11. Kumar L, M EF. Mfuzz: a software package for soft clustering of microarray data. Bioinformation. 2007;2:5–7; doi: 10.6026/97320630002005.
12. Langfelder P, Horvath S. WGCNA: an R package for weighted correlation

network analysis. BMC Bioinformatics. 2008;9:559; doi: 10.1186/1471-2105-9-559.

13. Watts SC, Ritchie SC, Inouye M, Holt KE. FastSpar: rapid and scalable correlation estimation for compositional data. Bioinformatics. 2019;35:1064–6; doi: 10.1093/bioinformatics/bty734.

14. Kang M-J, Bae E-K, Park E-J, Ka K-H, Son M-R, Kim K-T, et al. Draft genome sequence for the symbiotic pine mushroom *Tricholoma matsutake*. Microbiol Resour Ann. 2023;12:e01271–22; doi: 10.1128/mra.01271-22.

15. Smit A, Hubley R, Green P: RepeatMasker Open-4.0. In. Seattle, USA; 2015. https://www.repeatmasker.org/. Accessed 7 February 2025

16. Leinonen R, Sugawara H, Shumway M, Collaboration INSD. The sequence read archive. Nucleic Acids Res. 2010;39:D19–D21; doi: 10.1093/nar/gkq1019.

17. Kim D, Langmead B, Salzberg SL. HISAT: a fast spliced aligner with low memory requirements. Nat Methods. 2015;12:357–60; doi: 10.1038/nmeth.3317.

18. Li H, Handsaker B, Wysoker A, Fennell T, Ruan J, Homer N, et al. The sequence alignment/map format and SAMtools. Bioinformatics. 2009;25:2078–9; doi: 10.1093/bioinformatics/btp352.

19. Brůna T, Hoff KJ, Lomsadze A, Stanke M, Borodovsky M. BRAKER2: automatic eukaryotic genome annotation with GeneMark-EP+ and AUGUSTUS supported by a protein database. NAR Genom Bioinform. 2021;3:lqaa108; doi: 10.1093/nargab/lqaa108.

20. Lomsadze A, Burns PD, Borodovsky M. Integration of mapped RNA-Seq reads into automatic training of eukaryotic gene finding algorithm. Nucleic Acids Res. 2014;42:e119; doi: 10.1093/nar/gku557.

21. Stanke M, Schöffmann O, Morgenstern B, Waack S. Gene prediction in eukaryotes with a generalized hidden Markov model that uses hints from external sources. BMC Bioinformatics. 2006;7:62; doi: 10.1186/1471-2105-7-62.

22. Quinlan AR, Hall IM. BEDTools: a flexible suite of utilities for comparing genomic features. Bioinformatics. 2010;26:841–2; doi: 10.1093/bioinformatics/btq033.

23. Delgado LF, Andersson AF. Evaluating metagenomic assembly approaches for biome-specific gene catalogues. Microbiome. 2022;10:72; doi: 10.1186/s40168-022-01259-2.
